# Involvement of kynurenine pathway between inflammation and glutamate in the underlying etiopathology of CUMS-induced depression mouse model

**DOI:** 10.1186/s12868-022-00746-4

**Published:** 2022-11-10

**Authors:** Xingying Wu, Bowen Chen, Zhong Di, Shuo Jiang, Haipeng Xu, Mengting Shi, Rong Hu, Shaopeng Sun, Zhujin Song, Jiapeng Liu, Ruijie Ma, Qin Guo

**Affiliations:** 1 Department of Neurobiology and Acupuncture Research, The Third School of Clinical Medicine (School of Rehabilitation Medicine), Key Laboratory of Acupuncture and Neurology of Zhejiang Province, Zhejiang Chinese Medical University, Hangzhou, China; 2 Department of Acupuncture and Moxibustion, The Third Affiliated Hospital of Zhejiang Chinese Medical University, Hangzhou, 310000 People’s Republic of China; 3 Department of Acupuncture and Moxibustion, The First Affiliated Hospital of Zhejiang Chinese Medical University, Hangzhou, 310000 People’s Republic of China; 4 The First Clinical Medical College, Zhejiang Chinese Medical University, Hangzhou, 310053 China; 5 Basic Medical College, Zhejiang Chinese Medical University, Hangzhou, 310053 China

**Keywords:** Depression, Chronic unpredictable mild stress, Inflammation, Glutamate, Kynurenine pathway

## Abstract

Inflammation and glutamate (GLU) are widely thought to participate in the pathogenesis of depression, and current evidence suggests that the development of depression is associated with the activation of the kynurenine pathway (KP). However, the exact mechanism of KP among the inflammation, GLU and depression remain poorly understood. In this study, we examined the involvement of KP, inflammation and GLU in depressive phenotype induced by chronic unpredictable mild stress (CUMS) in C57B/6 J mice. Our results showed that CUMS caused depressive like-behavior in the sucrose preference test, tail suspension test and forced swimming test. From a molecular perspective, CUMS upregulated the peripheral and central inflammatory response and activated indoleamine 2,3-dioxygenase (IDO), the rate-limiting enzyme of KP, which converts tryptophan (TRP) into kynurenine (KYN). KYN is a precursor for QA in microglia, which could activate the N-methyl-D-aspartate receptor (NMDAR), increasing the GLU release, mirrored by increased IDO activity, quinolinic acid and GLU levels in the hippocampus, prefrontal cortex and serum. However, intervention with IDO inhibitor 1-methyl-DL-tryptophan (50 mg/kg/s.c.) and 1-methyl-L-tryptophan (15 mg/kg/i.p.) reversed the depressive-like behaviors and adjusted central and peripheral KP’s metabolisms levels as well as GLU content, but the inflammation levels were not completely affected. These results provide certain evidence that KP may be a vital pathway mediated by IDO linking inflammation and glutamate, contributing to depression.

**Supplementary Information:**

The online version contains supplementary material available at 10.1186/s12868-022-00746-4.

## Introduction

Depression is a recurrent mental disorder with a high prevalence in current times [1–3]. As of January 2020, the depressive disorder has developed into a disease that impacts over 264 million people of all ages worldwide [4]. In brief, depression is characterized by low mood, cognitive deficits, anhedonia, and even a high suicidal tendency [5, 6]. Over the years, numerous causes of depression have been identified, involving hypothalamic–pituitary–adrenal axis disorders, monoamine, neurotrophic factors, oxidative stress, cytokines, oxidative stress, neurotransmitter receptors, inflammation, and so on [7, 8]. Lately, the kynurenine pathway has been reported to play a key role. At present, the pharmacological treatment of major depression is often suboptimal and associated with substantial side effects [9–12]. Therefore, it is essential to identify the exact mechanisms of depression.

An increasing body of evidence suggests that inflammation is tightly related to stress and depression [13–15], depression and inflammation fuel one another [16]. Patients with depression have increased pro-inflammatory cytokines in the blood, such as interleukin-6 (IL-6), interleukin-1β (IL-1β), tumor necrosis factor-α (TNF-α) and other acute-phase proteins and C-reactive protein (CRP) [17, 18]. Moreover, accumulating evidence suggests that chronic stress can lead to exaggerated or prolonged inflammatory responses, resulting in a series of sickness behaviors (such as pain and disturbed sleep) and depressive symptoms (lack of pleasure) and act as mediating pathways prompting further severe inflammation and depression.

In addition, the levels of brain major excitatory and inhibitory neurotransmitters are related to the variation in brain connectivity in major depression disorder (MDD) [19], and glutamate (GLU), has an essential role in the central nervous system as the predominant excitatory neurotransmitter [20]. In neuroimaging and post-mortem studies, the glutamate levels of most depressed patients’ were elevated in the plasma, cerebrospinal fluid and brain [21, 22]. Therefore, dysregulation of glutamatergic neurotransmission is related to depressive-like behaviors [23, 24]. Moreover, our previous studies confirmed that increased glutamate concentration in the hippocampus (HIP) of depressed rats leads to neurotoxicity and apoptosis of neurons and astrocytes [25], and chronic stress leads to structural and morphological alternations such as dendritic spine loss, dendritic atrophy, and volume loss in the HIP and prefrontal cortex (PFC) [26, 27]

What's more, the kynurenine pathway (KP) plays an essential role in tryptophan degradation mediated by indoleamine 2,3-dioxygenase (IDO), an initial and rate-limiting enzyme. Given that the terminal metabolites of KP all act on N-methyl-D-aspartate (NMDA) receptors, the dysregulated activity of quinolinic acid (QA) and kynurenic acid (KYNA) on NMDA receptors and the hyperactivity of the glutamate system may be the critical point of KP’s involvement in depression. When activated, KP can induce the metabolism of TRP to shift towards kynurenine (KYN) and eventually degrades to QA and KYNA, impacting GLU neurotransmission at NMDA glutamate receptors, the former as an agonist at the glutamate binding site of the NMDA receptor, leading to excess GLU release which is increasingly implicated in neurodegenerative disorders, while the latter blocks the glycine-site of NMDA, which is a standard for the identification of glutamate-releasing synapses [28–30], endowing with neuroprotective agents [31–34].

KP is easily activated during inflammation, which is among the main pathological characteristics of depression and alters glutamate metabolism. Interestingly, IDO has also emerged as a potential target for depression treatment. However, the mechanism of KP linking glutamate and inflammatory factors to depression remains obscure and requires further investigation. Therefore, we are focusing on the questions of ‘how’ (pathways) and ‘for whom’ (predispositions) these links exist.

## Materials and methods

### Animals

Male C57BL/6 J mice (8–10 weeks; 20–25 g) with the approval number 20210315–15 were housed in the Laboratory Animal Center with access to food and water provided ad libitum. Standard laboratory conditions (12 h light: 12 h dark cycle, lights on at 08:00 pm, T = 21 ± 1 °C) were maintained in animal housing facilities. At the end of the tests, serum was collected after overnight fasting via eyeball blood while mice were anesthetized with Isoflurane. Data collected were subjected to statistical analysis with at least 5 mice per group.

### Experimental design in chronic unpredictable mild stress (CUMS) procedure

**Fig. 1:**
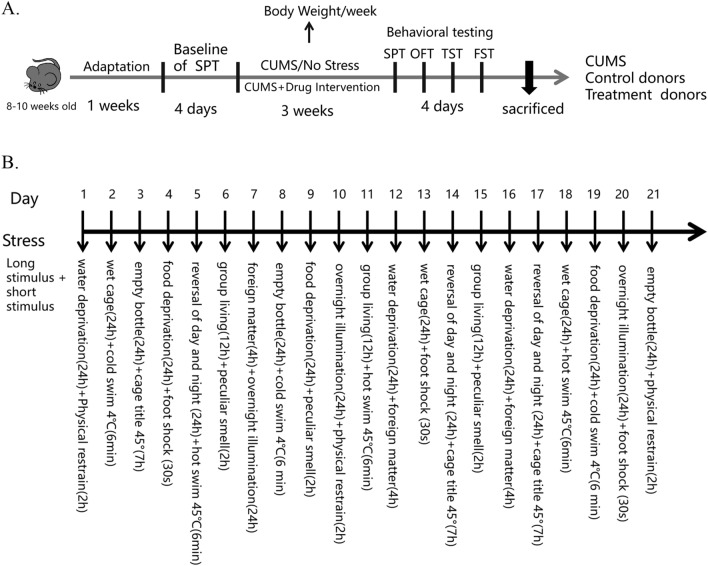
Experimental design. **A** Experimental timeline for the animals in the study. **B** The detailed protocol in the process of establishing a mice model of depression. The animals were habituated for 7 days and subjected to chronic unpredictable mild stress or administered with 1-MT for 21 days. Inhibitors were intraperitoneally or subcutaneously injected, and the behaviors were tested. Subsequently, brain tissue and serum were collected

After 1 week of adaptive feeding, mice were assigned to four groups, including the Control + PBS group, CUMS + PBS group, CUMS + DL-1MT group and CUMS + L-1MT group. Unstressed mice were group-housed in standard laboratory cages while the other groups were housed in individual cages, the latter were subjected to unpredictable mild stress protocol for 3 weeks. Two different stressors and sequences were applied each day. The protocol consisted of fourteen stressors as shown in Fig. 1B. After 3 weeks of the CUMS exposure, the behavioral approaches were performed to whether the model was successfully established. The body weights of the mice were measured weekly before and after the CUMS. The timeline of the experimental protocol is depicted in Fig. 1A.

### Drugs

1. Control + PBS and CUMS + PBS group received phosphate buffer solution (PBS, pH 7.4, without Ca2^+^ and Mg,2^+^) daily.
2. The IDO inhibitor, 1-methyl-D, L-tryptophan (DL-1-MT, Sigma-Aldrich, USA) was subcutaneously injected with a 5 ml/kg volume to deliver a dose of 50 mg/kg. The injections were administered twice daily with a 12-h interval between two administrations, and the effect is equivalent to the studies using 5 mg/day pellets [32]. The final pH was adjusted to 9.0 using 0.1 M NaOH.
3. The IDO inhibitor, 1-methyl-L-tryptophan (L-1-MT, Sigma-Aldrich, USA) was intraperitoneally injected for 21 d at 15 mg/kg/d. The drug was dissolved in 0.1 M sodium hydroxide and the pH was adjusted to 9.0 using hydrochloric acid in a volume ratio of 1:1 before administration [35].

### Behavioral analyses

#### Body weight measurement

Each mouse’s body weight was evaluated with an electronic balance at 9:00 am every Thursday.

#### Sucrose preference test (SPT)

The SPT was divided into training (as a baseline measurement) and testing periods. In brief, single-housed animals were trained with 1% sucrose solution for 24 h. Then after 24 h of food and water deprivation, all mice were provided with a bottle of pure water and another bottle of 1% sucrose solution simultaneously. Two hours later, the volumes of the remaining pure water and sucrose solution were recorded. It is defined as follows: sucrose preference percentage (%) = sucrose solution consumption (g)/(sucrose solution consumption [g] + water consumption [g]) × 100%.

#### Open-field test (OFT)

During the open filed test, mice were transported to the test room at least 2 h before the experiment for habituation. The room was kept quiet and the mice were placed in the center and allowed to move freely. The mice's behaviors were recorded for five minutes using a video camera mounted above the maze, which was analyzed using ANYmaze software (Stoelting). The time spent in the central area and total distance traveled were recorded automatically. After each session, the arena walls and floors were thoroughly wiped down with 75% ethanol to eliminate odor cues.

#### Tail-suspension test (TST)

During the tail-suspension test, mice were isolated and suspended by the tail taped on a stand at the edge of the tabletop 35 cm above [36]. The 6 min test was recorded and time spent immobile was measured during the last 4 min of the test.

#### Forced swimming test (FST)

During the forced swimming test, mice were placed in a transparent cylinder (30 cm height × 16 cm diameter, 14 cm of water depth, 24 °C) and they could neither touch the bottom nor climb out the top. Mice were forced to swim for 6 min. Consistent with TST, the animals were adapted for the first 2 min and the time spent immobile was recorded over the next 4 min.

### Quantitative real-time PCR analysis

Twenty-four hours after the final behavioral test, mice were sacrificed by rapid decapitation. Total RNA was extracted from brain tissue using TRIzol reagent (Invitrogen, USA), according to the manufacturer's instructions, cDNA Synthesis was performed the Prime Script First Strand Kit (Takara Biotechnology). The cDNA was amplified by PCR using standard methods. The following specific primers were used (see Table 1).

**Table 1:** Sequences of the primers used for qPCR

Sequence name	Primers’ sequence (5′to 3′)	Amplicon size (bp)
Actin	F: CACCCGCGAGTACAACCTTC R: CCCATACCCACCATCACACC	207
IL-1β	F:CAACTGTTCCTGAACTCAACTG R:GAAGGAAAAGAAGGTGCTCATG	290
TNF-α	F: GATCGGTCCCAACAAGGAGG R: GCTTGGTGGTTTGCTACGAC	138
IL-6	F: AGAGACTTCCAGCCAGTTGC R: CTGGTCTGTTGTGGGTGGTA	115
IDO1	F: CGAGAACATGGACATTCTGTTC R: TTTCCAATGCTTTCAGGTCTTG	316
IDO2	F:TGGATGGAAATTGCCCTCAGACTTC R:CGCTGCTCACGGTAACTCTTTAGG	232

### Enzyme-linked immunosorbent assay (ELISA)

Twenty-four hours after the last behavioral test, mice were sacrificed by decapitation. Brain tissue and serum were dissected. After naturally clotting for more than 20 min, the blood was centrifuged for 20 min (3000 rpm) and the supernatant was collected in the following experiment. Weights were taken of prefrontal cortex and hippocampal tissue, which was placed in a PBS solution (1 mL/10 mg) and homogenized rapidly. The samples of tissue and serum were centrifuged (5000 × *g* for 5 min), then the supernatant was collected and quantified. On the microplate reader, absorbance was quickly determined at 450 nm. And according to the standard curve, the levels of inflammatory cytokines IL-1β(JL18442), IL-6(JL20268) and TNF-α(JL10484), and KP enzyme IDO1(JL47052), IDO2(JL33530) concentrations were calculated by commercial ELISA kits (Jianglai, Shanghai, China) according to the manufacturer's protocol.

### High-performance liquid chromatography analysis (HPLC)

To understand the impact of stress on KP and its metabolic enzymes, the brain tissue and serum concentrations of tryptophan (TRP) and its IDO-catalyzed metabolite KYN、KYNA、QA were measured by HPLC and individual metabolite peaks detected were collected as HPLC fractions. The analysis was performed on an HPLC-1100 system (Agilent Technologies, USA) equipped with a quaternary pump and a UV detector for TRP and KYN, a fluorescence detection was used for measure KYNA and QA. HPLC analysis of the samples was performed using an Agilent C18 column (5 μm particle size, L × I.D. 25 cm × 4.6 mm) preceded by a C18 guard column (Agilent Technologies, USA). For TRP and KYN, the mobile phase was 15 mM acetic acid-sodium acetate buffer (pH 5.3) containing 6% acetonitrile by volume [37]. For KYNA and QA, Water and methanol with acetonitrile formate (0.1%) and formic acid (0.1%v/v) were used as mobile phase solvents [38].

### Statistical analysis

Statistical analyses were performed using GraphPad Prism 8 (GraphPad Software, San Diego, CA, USA). Moreover, our sample sizes were similar to those reported in previous publications. The data were expressed as the mean ± SEM and compared by a two-sided Student’s-*t*-test or one-way analysis of variance (ANOVA) followed by Bonferroni tests. Body weight and changes were analyzed via a repeated measures two-factor ANOVA. A *p*-value < 0.05 was statistically significant.

## Results

### CUMS induced depressive-like behavior

**Fig. 2:**
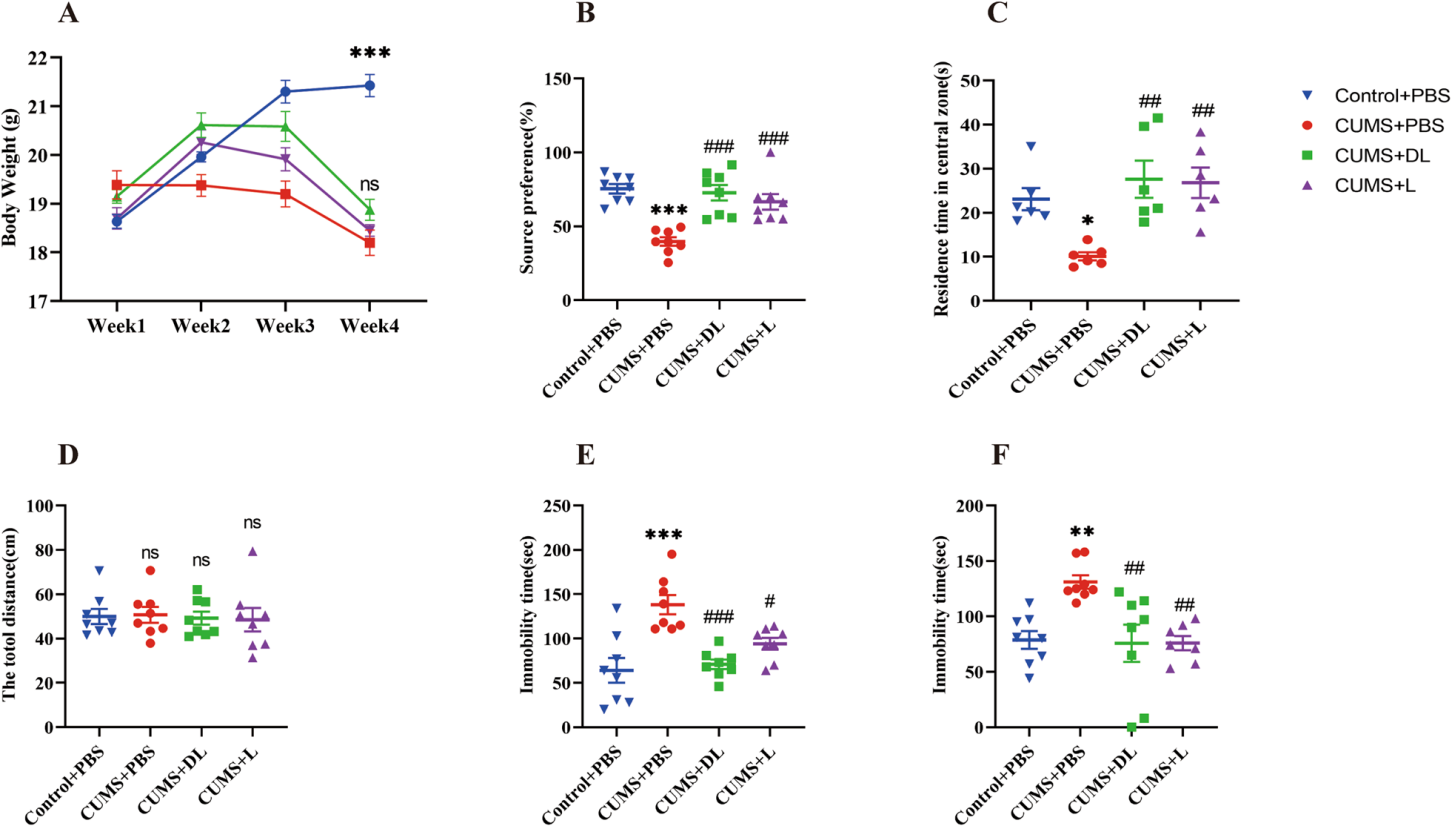
C57BL/6 J mice exhibited alterations in depression-like behaviors. **A** Results of the body weight of mice after a 3-week exposure to CUMS (mean ± SEM, *n* = *6- 8*). **B** Results of sucrose preference of mice after a 3-week exposure to CUMS. **C** Results of central time in OFT test of mice after a 3-week exposure to CUMS. **D** Results of total distance in OFT test of mice after a 3-week exposure to CUMS. **E** Results of tail suspension test of mice after a 3-week exposure to CUMS. **F** Results of forced swimming test of mice after a 3-week exposure to CUMS. Data are presented as mean ± SEM. *p < 0.05, **p < 0.01, ***p < 0.001, ****p < 0.0001 vs Control + PBS, ^*#*^*p* < 0.05, ^*##*^*p* < 0.01, ^*###*^*p* < 0.001, ^*####*^*p* < 0.0001 vs CUMS + PBS

Mice underwent 3 weeks of CUMS exposure, then were weighed and tested for sickness or depression-like behavior before being euthanized to collect brain samples and serum. The CUMS-induced sickness was measured on the first day of week one by assessing body weight loss. (Fig. 2A) and one day after the final stress session by assessing the sucrose solution consumption (Fig. 2B). To investigate CUMS-induced depressive-like behaviors, all mice underwent behavioral tests including OFT, TST, and FST (Fig. 2C–F). As expected, there was a significant difference in body weight change after CUMS (*p* < 0.0001). Similarly, the sucrose consumption of CUMS-exposed mice was significantly reduced (*p* < 0.0001), which reflected anhedonia (Fig. 2B). After CUMS exposure, mice spent less time on the center zone than controls during the OFT test (*p* < 0.05) (Fig. 2C), however, the distance traveled in the open field was comparable among the groups, which indicated that mice's motor function was not impaired (*p* > 0.05) (Fig. 2D). The mice exposed to CUMS exhibited increased time spent immobile in TST (*p* < 0.01) (Fig. 2D) and FST (*p* < 0.0001) (Fig. 2E) compared to the control mice. These results indicate that mice exposed to CUMS displayed significant depressive-like behaviors,

### CUMS induces central and peripheral cytokine expression

**Fig. 3:**
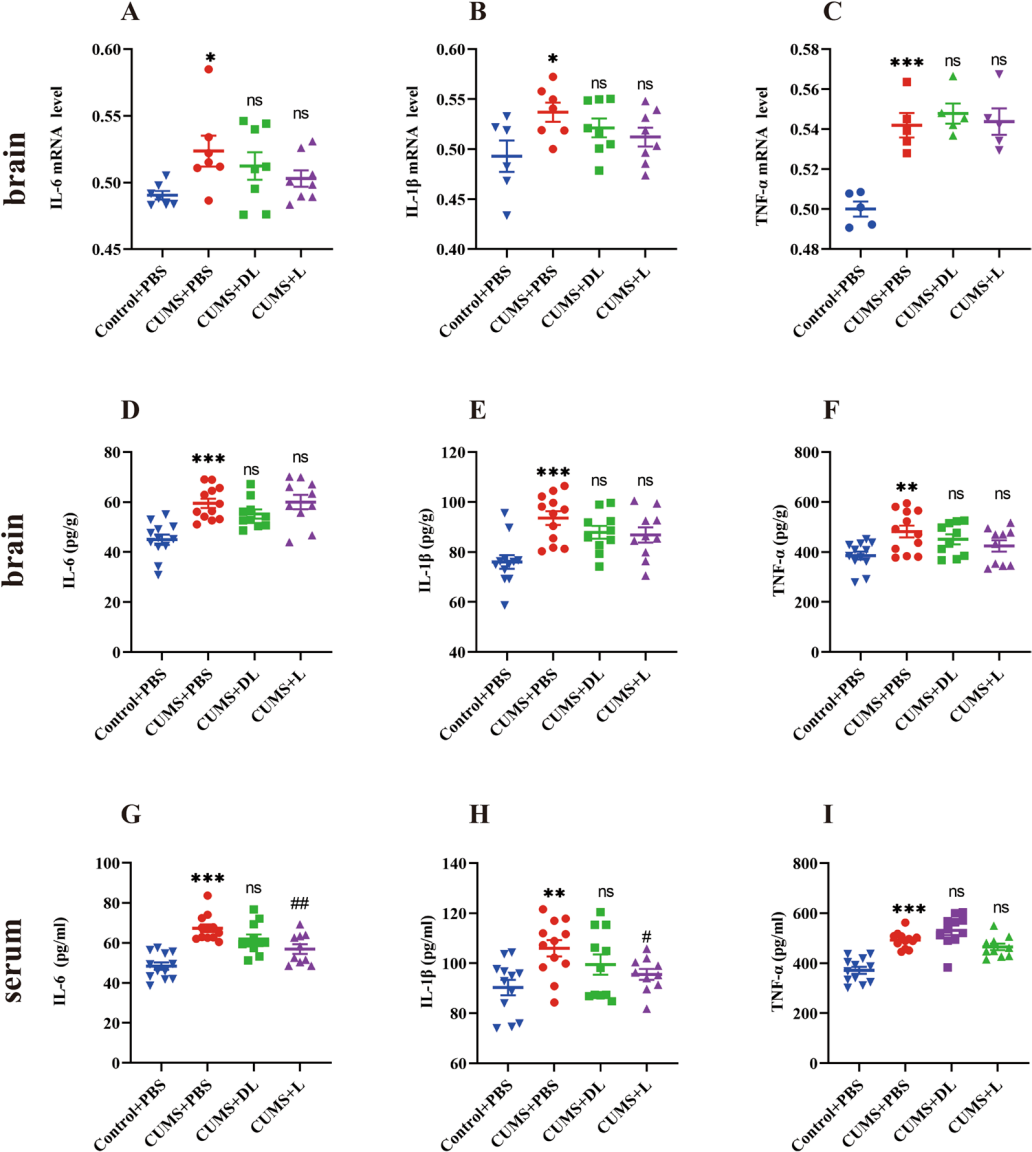
CUMS mice activate cytokines. **A**–**I** Results of the IL-6, IL-1β and TNF-α of mice after a 3-week exposure to CUMS through qPCR and ELISA. Data are presented as mean ± SEM, *n* = *5–8 per group*. *p < 0.05, **p < 0.01, ***p < 0.001, ****p < 0.0001 vs Control + PBS, ^*#*^*p* < 0.05*, *^*##*^*p* < 0.01, ^*###*^*p* < 0.001*, *^*####*^*p* < 0.0001 vs CUMS + PBS

To validate that our model was successful and verify the relationship between central and peripheral cytokines and depression, we measured the mRNA expression and protein level of pro-inflammatory cytokines, including IL-6, IL-1β and TNF-α. As shown in Fig. 3A–C, CUMS increased the expression of IL-6 (*p* < 0.05), IL-1β (*p* < 0.05) and TNF-α (*p* < 0.001) mRNA compared with the Control + PBS group. Meanwhile, our data showed that stress stimuli dramatically increased the levels of IL-6 (*p* < 0.01), IL-1β (*p* < 0.0001) and TNF-α (*p* < 0.01) (Fig. 3D–I) in the brain and serum.

### CUMS increased the release of GLU in mouse serum and brain

GLU, is a major excitatory neurotransmitter, that plays a vital role in the central nervous system [20]. However, the changes in GLU levels in depressed mice serum and brain remain poorly understood. We observed central and peripheral GLU levels by HPLC and found that GLU was a significantly elevated in the serum of CUMS mice model (Fig. 5J) (*p* < *0.01*). In contrast, GLU was elevated in brain tissues in the model group but did not differ from control mice (*p* > *0.05*) (Fig. 5D).

### CUMS activated IDO and mediated KP and its metabolism

**Fig. 4:**
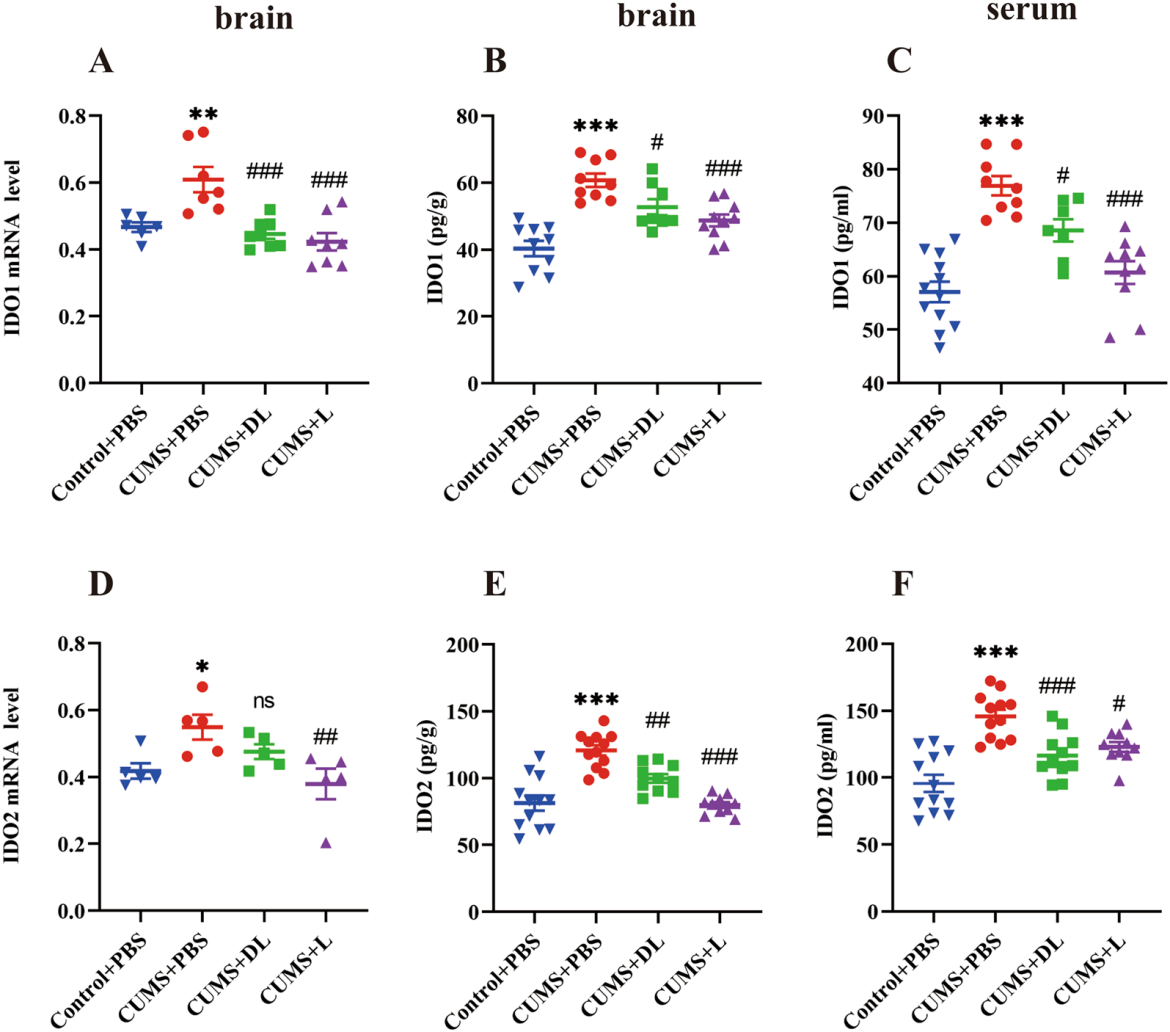
CUMS mice activate IDO and cytokines. **A**–**F** Results of the IDO1 and IDO2 of mice after a 3-week exposure to CUMS through qPCR and ELISA. Data are presented as mean ± SEM, *n* = *5–8 per group*. *p < 0.05, **p < 0.01, ***p < 0.001, ****p < 0.0001 vs Control + PBS, ^*#*^*p* < 0.05*, *^*##*^*p* < 0.01, ^*###*^*p* < 0.001*, *^*####*^*p* < 0.0001 vs CUMS + PBS

Given that IDO is the first rate-limiting and inflammatory-inducing enzyme in the KP [39], we explored the molecular mechanisms underlying stress-induced depression. Accordingly, we measured two IDO isoforms, IDO1and IDO2, KP and its metabolites in the serum and brain tissue of mice harvested at the end of behavioral experiments by q-PCR, ELISA and HPLC. We found that brain (Fig. 4A, B, D, E) and serum (Fig. 4C, F) expression of IDO1 and IDO2 were significantly increased in CUMS induced-mice (*p* < *0.01*), indicating that CUMS-exposure elevated IDO expression in mice.

**Fig. 5:**
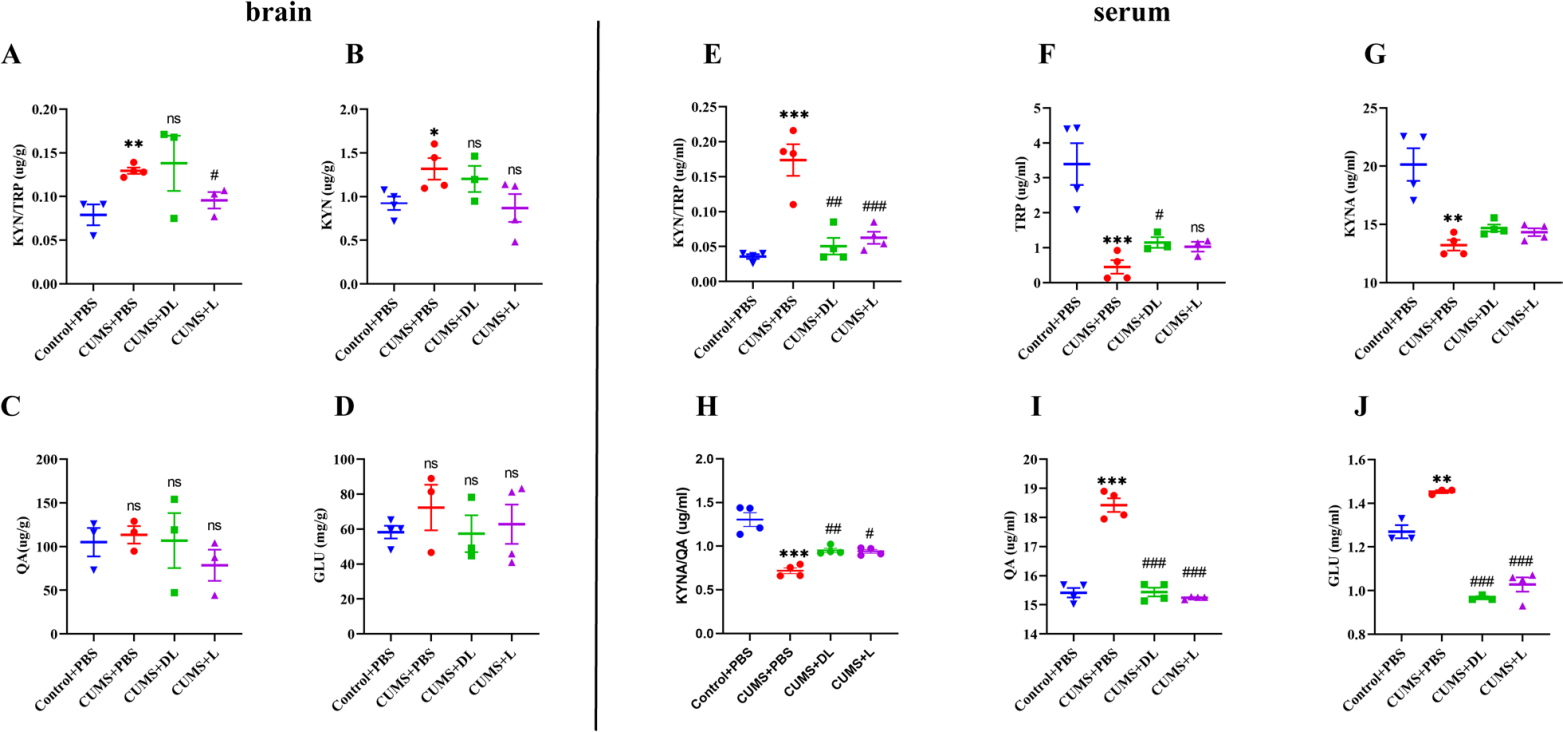
The changes in KP and its metabolites under the stress. **A**–**D** The level of KYN/TRP (**A**), KYN (**B**), QA (**C**) and GLU (**D**) in the brain were measured via HPLC. **E**–**I** The results of KYN/TRP (**E**), TRP (**F**), QA (**G**), KYNA/QA (**H**), KYNA (**I**) and GLU (**J**) in the serum. Data are expressed as the mean ± SEM. ** p < 0.01, ****p < 0.0001 *vs.* Control group; ^*#*^*p* < 0.05, ^*##*^*p* < 0.01, ^*###*^*p* < 0.001, ^*####*^*p* < 0.0001 vs model

In addition to IDO, other downstream enzymes in the KP—including TRP, KYN, KYNA and QA, were analyzed in the CUMS group. As expected, KYN/TRP was an indicator of IDO activity [40], we found the ratio of KYN to TRP was increased in the brain and serum (Fig. 5A, E) (*p* < *0.01*), suggesting it has a significant value as a surrogate marker of IDO activation [32, 41]. Moreover, the level of KYN was increased (*p* < *0.05*) (Fig. 5B) while QA exhibited no significant change (*p* > *0.05*) (Fig. 5C) during HPLC. However, the KP exhibited significant changes in the peripheral region, the level of TRP and KYNA was decreased (*p* < *0.001*) (Fig. 5F, G) while QA was elevated (*p* < *0.0001*) (Fig. 5I). Of note, there was a difference observed for KYNA/QA ratio (Fig. 5H) (*p* < *0.001*), a measure of NMDA agonist/antagonist balance. These findings established that IDO could be activated by stress and then mediated the change in KP. To better verify these results, we also performed the correlation analysis between the level of QA and GLU, QA and cytokines respectively (see Additional file 1: Fig. S1, Table S1).

### 1-MT specifically inhibits IDO and depressive-like behavior

To directly target IDO for in vivo experiments, mice were injected with DL-1-MT (50 mg/kg) or L-1-MT (15 mg/kg) for 3 weeks. First, we verified the inhibitory effect on IDO1 and IDO2 by q-PCR, as shown in Fig. 4A and D, respectively, 1-MT specifically inhibited the mRNA expression and protein level of IDO1 (*p* < *0.001*) and IDO2 (*p* < *0.01*); and through HPLC, we found that L-1-MT attenuated the increased ratio of kynurenine to tryptophan in the brain (Fig. 5A) (*p* < *0.05*) and peripheral blood (Fig. 5E) (*p* < *0.001*), however, DL-1-MT only affected the peripheral ratio value (Fig. 5E) (*p* < *0.01*), and decreased the level of TRP in the serum (Fig. 5F) (*p* < *0.05*). What’s more, the ratio of KYNA/QA as well as the concentration of QA (Fig. 5H, I) could only be reversed by two inhibitors, suggesting the KP was activated strongly in the peripheral blood and propelled kynurenine transport from the blood to the brain, which agreement with the Robert Danzer’s results [42].

Depressive-like behaviors were also measured post-1-MT injection. In contrast with mice treated with PBS, the 1-MT intervention did not affect the CUMS-induced reduction in body weight (Fig. 2A) (*p* > *0.05*). However, injection with DL-1-MT (*p* < *0.0001*) or L-1-MT (*p* < *0.001*) significantly reversed the decrease in sucrose preference percentage induced by CUMS (Fig. 2B), increased the time spent in the center area during the open field test (Fig. 2D) (*p* < *0.01*), and reduced the time spent during TST (*p* < *0.01*) and FST (*p* < *0.001*) compared with the CUMS group (Fig. 2E, F). Overall, these findings indicated that IDO inhibitors could ameliorate depressive-like behavior (Additional file 2, 3, 4).

### 1-MT blocks peripheral CUMS-induced cytokine and GLU content

Then, to determine whether DL-1-MT or L-1-MT yielded an anti-inflammatory effect, IL-6, IL-1β and TNF-α mRNA expression (Fig. 3A–C) and protein levels (Fig. 3D–I) were measured in the brains and serum in mice exposed to CUMS. Our results found that 1-MT did not modify the levels and mRNA expression of cytokines; however, as shown in Fig. 3GH, L-1-MT inhibited the protein level of IL-6 (*p* < *0.01*) and IL-1β (*p* < *0.05*), suggesting L-1-MT had a definite anti-inflammatory effect.

We also assessed the function of inhibitors on GLU and found that DL-1-MT or L-1-MT significantly decreased GLU levels in the serum (Fig. 5J) (*p* < *0.001*) than in brain tissues (Fig. 5D) (*p* > *0.05*), showing the inhibitors had a more significant effect on peripheral GLU levels.

## Discussion

Two evolving theories about the development of mood disorders involve hyperactivation of inflammatory cytokines and altered glutamate metabolism [43–45]. Stress can cause depression, perhaps partly because it propels inflammation and then alters glutamate metabolism [46–48], and KYN metabolism has been identified as a critical neurochemical pathway linking inflammation and depression [49], which also regulates the release of glutamine. Therefore, in our study, we established a CUMS mice model and, observed alleviation of depression via changes in the behavioral test, glutamate and proinflammatory factor levels; we explored whether associations between inflammation and glutamine in depression were associated with the kynurenine pathway.

It is well-established that inflammation is linked to depression. An increasing body of evidence from recently published, human studies has associated increased peripheral and central cytokines production with the development of mood disorders [50, 51]. This increased activation of inflammatory, in turn, contributes to the glutamatergic system’s pathological activation, which leads to excitotoxicity and decreased neurotrophic support in the CNS. In fact, the dysfunction of the immune, monoaminergic, and glutamatergic systems is implicated in the pathology of depression. KP is a potential intersection of these systems, where immune responses and serotonergic neurotransmission are catabolized by TRP to KYN, ultimately altering downstream synaptic glutamate neurotransmission [52]. Enhanced pro-inflammatory cytokine levels may over-activate the KP, leading to tryptophan depletion and reduced serotonin levels, which can subsequently precipitate depressive symptoms, however, only a small fraction of the TRP pool is converted to serotonin, the vast majority (~ 95%) is metabolized via KP [53].

Apart from depression and inflammation, altered glutamate metabolism also implicates mood disorders [54–56]. Dysregulation of glutamate release is one of the major triggers of depression and is involved in the mechanism of antidepressant effects [25, 43, 57] which is the most abundant amino acid in the blood and cerebrospinal fluid and is the precursor of major central nervous system stimulants [20]. QA was an excitotoxic kynurenine pathway metabolite of tryptophan, enhancing glutamate release and inhibiting glutamate uptake. In our research, increased levels of pro-inflammatory cytokines and GLU in peripheral and central regions made us focus attention on the relationship of inflammation and glutamate release to explore the mechanisms underlying depression-like behavior induced by CUMS. So we did a correlation analysis to better illustrate the relationship between QA and glutamate and inflammatory factors, and found QA had a strong positive correlation with GLU, while hardly related with IL-6, IL-1β and TNF-α in the brain. Meanwhile, we observed that the QA had a strong linear correlation with GLU, and a moderate positive correlation with IL-6 and IL-1β, a weak correlation with TNF-α in the serum, suggesting QA may act as a bridge between the peripheral cytokines and glutamate.

It is well-recognized that the relationship between inflammation and depression [32] is mediated by IDO activation which is an enzyme involved in kynurenine synthesis from tryptophan [58]. Importantly, the ratio of KYN/TRP is a proxy for IDO enzymatic activity [59]. Two isomers of IDO, IDO1 and IDO2, have been reported to participate in TRP conversion into KYN and other downstream metabolites [60, 61]. Nonetheless, the roles of IDO1 and IDO2 in this mechanism remain obscure [60], especially IDO2. Since DL-1-MT, a mixture of two isomers including L-1-MT and D-1-MT, could inhibit both IDO1 and IDO2. And L-1-MT is a more potent inhibitor of IDO1. We wanted to test which isomer could exert its observed effect on depression via this route. And we found that IDO1 and IDO2 activity were elevated both peripherally and centrally during the stress and changed the depressive-like behaviors. However, L-1-MT, a competitive inhibitor of IDO1, significantly lower the ratio of KYN/TRP, reversed the level of IL-6 and IL-1β in serum, possessing anti-inflammatory effects, which was consistent with the literature [62, 63]. It has been established that IDO2 plays a cytokine suppressor role while IDO1 is a pro-inflammatory one [63–65], and L-1-MT can block IDO1. In our study, L-1-MT suppressed the levels of IL-6 and IL-1β. Besides, DL-1-MT, a racemic compound inhibitor of IDO1 and IDO2 [62, 66, 67], yielded no significant effect on the proinflammatory cytokines.

In addition to IDO, KP and its metabolites have been reported to link inflammation and depression through effects on brain glutamate receptors. Increased glutamate in inflammatory conditions results from the effect of inflammatory cytokines on IDO [68, 69]. The KP is an alternative but distinct pathway to the generation of glutamate receptor ligands. The products of tryptophan metabolism via the kynurenine pathway include both quinolinic acid and kynurenic acid. As an agonist of NMDA receptors, QA leads to increased potential neurotoxic metabolites and releases large amounts of glutamate [70], and KYNA, a potentially neuroprotective compound [71], that aggravates depression at low levels [72, 73]. In addition to affecting excitatory neurotransmission by acting directly on glutamate receptors, QA and KYNA indirectly modulate glutamate function. For example, QA-induced stimulation of NMDA receptors in the rat cerebral cortex results in a substantial increase in glutamate release.

Previous studies have associated KP metabolites with the severity of depression including feelings of hopelessness and lack of motivation [49, 74]. Our data support these findings and suggest that the combined activation of inflammation and KP in the periphery may play an important role in depression, inflammation and GLU. Moreover, our data illustrated the specificity of IDO inhibitors, DL-1-MT and L-1-MT, which could reverse the depressive behaviors and may partly contribute to the release of inflammatory cytokines and GLU. In conclusion, this study describes a putative pathway that drives depression involving inflammatory mediators, GLU and KP metabolites. At last, there are also some limitations to this study. Firstly, in the behavioral experiment we did not include a placebo group as the objective was not to assess once more the effects of 1-MT but just to confirm that 1-MT did block the IDO-induced depression-like behaviors. Secondly, we only used male mice to verify our results and did not study female mice to examine the effect of KP on sex differences, which need further research. Thirdly, we ignore the tryptophan 2,3-dioxygenase (TDO), an enzyme with a similar function to IDO, which is capable of inducing stress-induced depression in the rat and driving the metabolism of tryptophan through the KP [38]. Despite these limitations, the conclusion still can be made that the kynurenine pathway involved the underlying etiopathology of CUMS between inflammation and glutamate.

## Conclusion

**Fig. 6:**
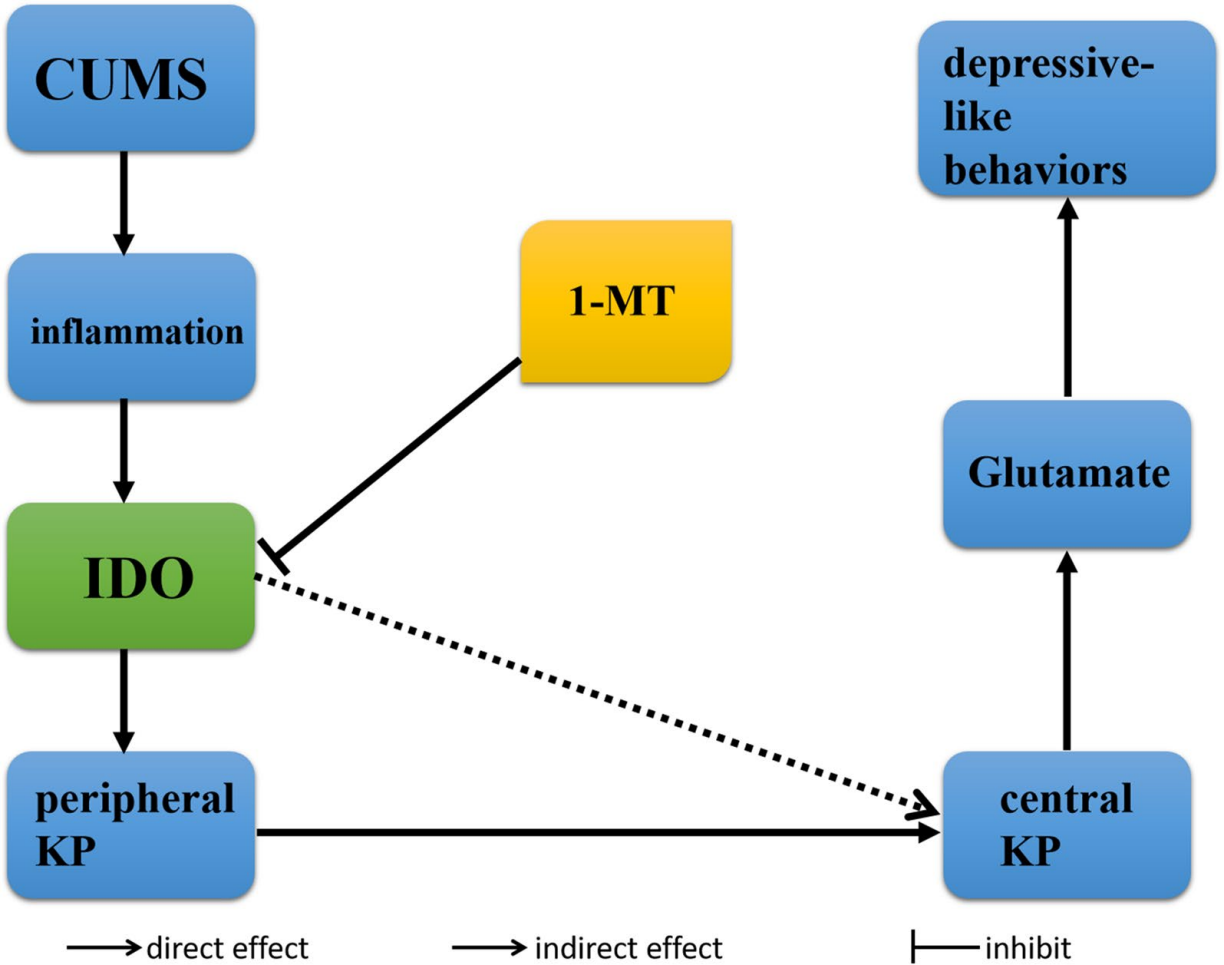
The directional associations of serum and brain kynurenine pathway (KP) among CUMS, inflammation, glutamate. The broken arrows represent indirect effects and the filled arrows represent direct effects, whereas truncated lines represent an inhibitory action or effect. CUMS = chronic mild unpredictable stress; KP = kynurenine pathway; 1-MT = 1-methyl-DL/L-tryptophan

A summary of our findings on the mechanisms of the kynurenine pathway linking inflammation and GLU in depression is provided in Fig. 6. These results corroborate that KP mechanisms may be used to prevent and treat CUMS-induced depression-like symptoms.

## Supplementary Information

**Additional file 1: Figure S1.** The correlation analysis between the level of QA and GLU, QA andAQ5 cytokines respectively. (A-E) The level of GLU(A), KYN/TRP(B), KYN(C) KYNA(D) and QA(E) in the brain were measured via HPLC. (F-I) The results of GLU(E), KYN/TRP(F), QA(G) and TRP(H) in the serum. Data are expressed as the mean ± SEM. ** p < 0.01, ****p < *0.0001 vs. control group; *^*#*^*p* < *0.05, *^*##*^*p* < *0.01, *^*###*^*p* < *0.001, *^*####*^*p* < *0.0001* vs CUMS. The Pearson correlation coefficients were used to evaluate the correlation of continuous variables, which are scaled such that they range from –1 to + 1, where 0 indicates that there is no linear or monotonic association, and the relationship gets stronger and ultimately approaches a straight line, what’s more, datas with correlation coefficient < 0.25 were evaluated as ‘weak’correlation, ≥ 0.25 and < 0.5 as ‘moderate’ correlation, ≥ 0.5 and < 0.75 as ‘strong’ and ≥ 0.75 as very strong correlation(PMID: 30672319).

**Additional file 2: Table S2.** The mean and SEM of metabolites for all the groups.

**Additional file 3: Table S3.** Lowest level of quantification and the intra-assay percentage of coefficient of variation for the analytes measured by high performance liquid chromatography with tandem mass spectrometry.

**Additional file 4.** The chromatogram of KYNA and QA.

## Data Availability

The data used and/or analyzed during the current study are available from the corresponding author on reasonable request.
