# Supplementary material for: Involvement of kynurenine pathway between inflammation and glutamate in the underlying etiopathology of CUMS-induced depression mouse model
Source: BMC Neurosci. 2022 Nov 10;23:62. doi: 10.1186/s12868-022-00746-4 (PMC9650798; doi:10.1186/s12868-022-00746-4)
Supplement: Supplementary file 1 — Additional file 1: Figure S1. The correlation analysis between the level of QA and GLU, QA andAQ5 cytokines respectively. (A-E) The level of GLU(A), KYN/TRP(B), KYN(C) KYNA(D) and QA(E) in the brain were measured via HPLC. (F-I) The results of GLU(E), KYN/TRP(F), QA(G) and TRP(H) in the serum. Data are expressed as the mean ± SEM. ** p < 0.01, ****p < 0.0001 vs. control group; #p < 0.05, ##p < 0.01, ###p < 0.001, ####p < 0.0001 vs CUMS. The Pearson correlation coefficients were used to evaluate the correlation of continuous variables, which are scaled such that they range from –1 to + 1, where 0 indicates that there is no linear or monotonic association, and the relationship gets stronger and ultimately approaches a straight line, what’s more, datas with correlation coefficient < 0.25 were evaluated as ‘weak’correlation, ≥ 0.25 and < 0.5 as ‘moderate’ correlation, ≥ 0.5 and < 0.75 as ‘strong’ and ≥ 0.75 as very strong correlation(PMID: 30672319). [file 12868_2022_746_MOESM1_ESM.docx]

**Figure S1**


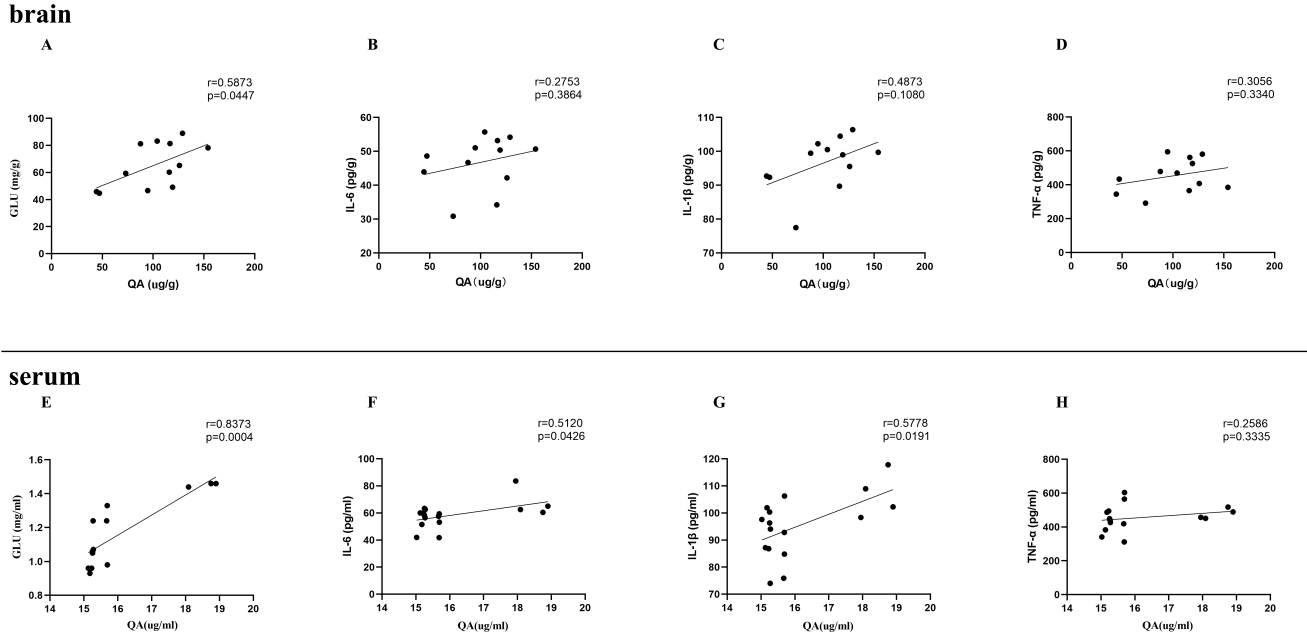


The correlation analysis between the level of QA and GLU, QA andAQ5 cytokines respectively. (A-E) The level of GLU(A), KYN/TRP(B), KYN(C) KYNA(D) and QA(E) in the brain were measured via HPLC. (F-I) The results of GLU(E), KYN/TRP(F), QA(G) and TRP(H) in the serum. Data are expressed as the mean ±SEM. *** p < 0.01, ****p < 0.0001 vs. control group; ^#^p < 0.05, ^##^p < 0.01, ^###^p < 0.001, ^####^p < 0.0001* vs CUMS.

**Table S1**

| QA | | |
| --- | --- | --- |
|  | brain | serum |
|  | r p | r p |
| GLU | 0.5873 0.0447 | 0.8373 0.0004 |
| IL-6 | 0.2753 0.3564 | 0.5120 0.0426 |
| IL-1β | 0.4873 0.1080 | 0.5778 0.0191 |
| TNF-α | 0.3056 0.3340 | 0.2586 0.3335 |

The Pearson correlation coefficients were used to evaluate the correlation of continuous variables, which are scaled such that they range from –1 to +1, where 0 indicates that there is no linear or monotonic association, and the relationship gets stronger and ultimately approaches a straight line, what’s more, datas with correlation coefficient ＜ 0.25 were evaluated as ‘weak’correlation, ≥0.25 and ＜0.5 as ‘moderate’ correlation, ≥0.5 and ＜0.75 as ‘strong’ and ≥ 0.75 as very strong correlation（PMID: 30672319）.

IDO: indoleamine 2,3-dioxygenase; TDO: tryptophan 2,3-dioxygenase; NAS: N -acetylserotonin.
