# Supplementary material for: Involvement of kynurenine pathway between inflammation and glutamate in the underlying etiopathology of CUMS-induced depression mouse model
Source: BMC Neurosci. 2022 Nov 10;23:62. doi: 10.1186/s12868-022-00746-4 (PMC9650798; doi:10.1186/s12868-022-00746-4)
Supplement: Supplementary file 2 — Additional file 2: Table S2. The mean and SEM of metabolites for all the groups. [file 12868_2022_746_MOESM2_ESM.docx]

**Table S2**

The mean and SEM of metabolites for all the groups

**Brain KYN/TRP**

|  | Control+PBS | CUMS+PBS | CUMS+DL | CUMS+L |
| --- | --- | --- | --- | --- |
| Mean | 0.07900 | 0.1295 | 0.1380 | 0.09567 |
| Std. Error of Mean | 0.01200 | 0.003524 | 0.03151 | 0.009404 |

**Brain KYN**

|  | Control+PBS | CUMS+PBS | CUMS+DL | CUMS+L |
| --- | --- | --- | --- | --- |
| Mean | 0.9240 | 1.318 | 1.202 | 0.8698 |
| Std. Error of Mean | 0.07685 | 0.1233 | 0.1490 | 0.1589 |

**Brain QA**

|  | Control+PBS | CUMS+PBS | CUMS+DL | CUMS+L |
| --- | --- | --- | --- | --- |
| Mean | 105.0 | 113.4 | 106.8 | 78.65 |
| Std. Error of Mean | 16.21 | 10.01 | 31.45 | 17.83 |

**Brain GLU**

|  | Control+PBS | CUMS+PBS | CUMS+DL | CUMS+L |
| --- | --- | --- | --- | --- |
| Mean | 58.23 | 72.31 | 57.33 | 62.80 |
| Std. Error of Mean | 3.604 | 13.04 | 10.53 | 11.25 |

**Serum KYN/TRP**

|  | Control+PBS | CUMS+PBS | CUMS+DL | CUMS+L |
| --- | --- | --- | --- | --- |
| Mean | 0.03575 | 0.1738 | 0.05050 | 0.06250 |
| Std. Error of Mean | 0.003326 | 0.02252 | 0.01184 | 0.008646 |

**Serum TRP**

|  | Control+PBS | CUMS+PBS | CUMS+DL | CUMS+L |
| --- | --- | --- | --- | --- |
| Mean | 3.396 | 0.4482 | 1.149 | 1.028 |
| Std. Error of Mean | 0.5975 | 0.1938 | 0.1512 | 0.1395 |

**Serum QA**

|  | Control+PBS | CUMS+PBS | CUMS+DL | CUMS+L |
| --- | --- | --- | --- | --- |
| Mean | 15.41 | 18.42 | 15.44 | 15.24 |
| Std. Error of Mean | 0.1611 | 0.2361 | 0.1501 | 0.02209 |

**Serum KYNA/QA**

|  | Control+PBS | CUMS+PBS | CUMS+DL | CUMS+L |
| --- | --- | --- | --- | --- |
| Mean | 1.304 | 0.7185 | 0.9522 | 0.9403 |
| Std. Error of Mean | 0.07784 | 0.03316 | 0.02367 | 0.02104 |

**Serum GLU**

|  | Control+PBS | CUMS+PBS | CUMS+DL | CUMS+L |
| --- | --- | --- | --- | --- |
| Mean | 1.270 | 1.453 | 0.9667 | 1.028 |
| Std. Error of Mean | 0.03000 | 0.006667 | 0.006667 | 0.03276 |

We have added the mean and SEM of the rest of metabolites and the negative results as shown below:

**Brain TRP**

|  | Control+PBS | CUMS+PBS | CUMS+DL | CUMS+L |
| --- | --- | --- | --- | --- |
| Mean | 11.96 | 9.460 | 10.86 | 9.282 |
| Std. Error of Mean | 0.6118 | 0.9607 | 1.137 | 1.495 |

**Brain KYNA**

|  | Control+PBS | CUMS+PBS | CUMS+DL | CUMS+L |
| --- | --- | --- | --- | --- |
| Mean | 75.16 | 81.45 | 53.16 | 45.73 |
| Std. Error of Mean | 1.998 | 13.90 | 14.01 | 9.240 |

**Serum KYN**

|  | Control+PBS | CUMS+PBS | CUMS+DL | CUMS+L |
| --- | --- | --- | --- | --- |
| Mean | 10.99 | 9.841 | 10.58 | 7.574 |
| Std. Error of Mean | 0.4571 | 0.6775 | 1.294 | 0.7570 |

**Serum KYNA**

|  | Control+PBS | CUMS+PBS | CUMS+DL | CUMS+L |
| --- | --- | --- | --- | --- |
| Mean | 20.14 | 13.21 | 14.69 | 14.33 |
| Std. Error of Mean | 1.405 | 0.4556 | 0.3029 | 0.3367 |
