# Supplementary material for: Involvement of kynurenine pathway between inflammation and glutamate in the underlying etiopathology of CUMS-induced depression mouse model
Source: BMC Neurosci. 2022 Nov 10;23:62. doi: 10.1186/s12868-022-00746-4 (PMC9650798; doi:10.1186/s12868-022-00746-4)
Supplement: Supplementary file 3 — Additional file 3: Table S3. Lowest level of quantification and the intra-assay percentage of coefficient of variation for the analytes measured by high performance liquid chromatography with tandem mass spectrometry. [file 12868_2022_746_MOESM3_ESM.docx]

**Table S3**

Lowest level of quantification and the intra-assay percentage of coefficient of variation for the analytes measured by high performance liquid chromatography with tandem mass spectrometry.

|  | **Batch 1** |  |
| --- | --- | --- |
| **Analyte** | LLOQ | % CV |
| KynA | 20 nM | 5.84 |
| QA | 45 nM | 4.70 |
|  | **Batch 2** |  |
| **Analyte** | LLOQ | % CV |
| KynA | 20 nM | 9.8 |
| QA | 45 nM | 7.98 |

Brain KYNA

|  | Control+PBS | CUMS+PBS | CUMS+DL | CUMS+L |
| --- | --- | --- | --- | --- |
| Mean | 75.16 | 81.45 | 53.16 | 45.73 |
| Std. Deviation | 3.460 | 24.07 | 24.26 | 16.00 |

Serum KYNA

|  | Control+PBS | CUMS+PBS | CUMS+DL | CUMS+L |
| --- | --- | --- | --- | --- |
| Mean | 20.14 | 13.21 | 14.69 | 14.33 |
| Std. Deviation | 2.810 | 0.9112 | 0.6058 | 0.6733 |

Brain QA

|  | Control+PBS | CUMS+PBS | CUMS+DL | CUMS+L |
| --- | --- | --- | --- | --- |
| Mean | 105.0 | 113.4 | 106.8 | 78.65 |
| Std. Deviation | 28.07 | 17.34 | 54.47 | 30.89 |

Serum QA

|  | Control+PBS | CUMS+PBS | CUMS+DL | CUMS+L |
| --- | --- | --- | --- | --- |
| Mean | 15.41 | 18.42 | 15.44 | 15.24 |
| Std. Deviation | 0.3222 | 0.4722 | 0.3002 | 0.04418 |
