# Supplementary material for: Involvement of kynurenine pathway between inflammation and glutamate in the underlying etiopathology of CUMS-induced depression mouse model
Source: BMC Neurosci. 2022 Nov 10;23:62. doi: 10.1186/s12868-022-00746-4 (PMC9650798; doi:10.1186/s12868-022-00746-4)
Supplement: Supplementary file 4 — Additional file 4. The chromatogram of KYNA and QA. [file 12868_2022_746_MOESM4_ESM.docx]

The chromatogram of KYNA and QA.

QA S1


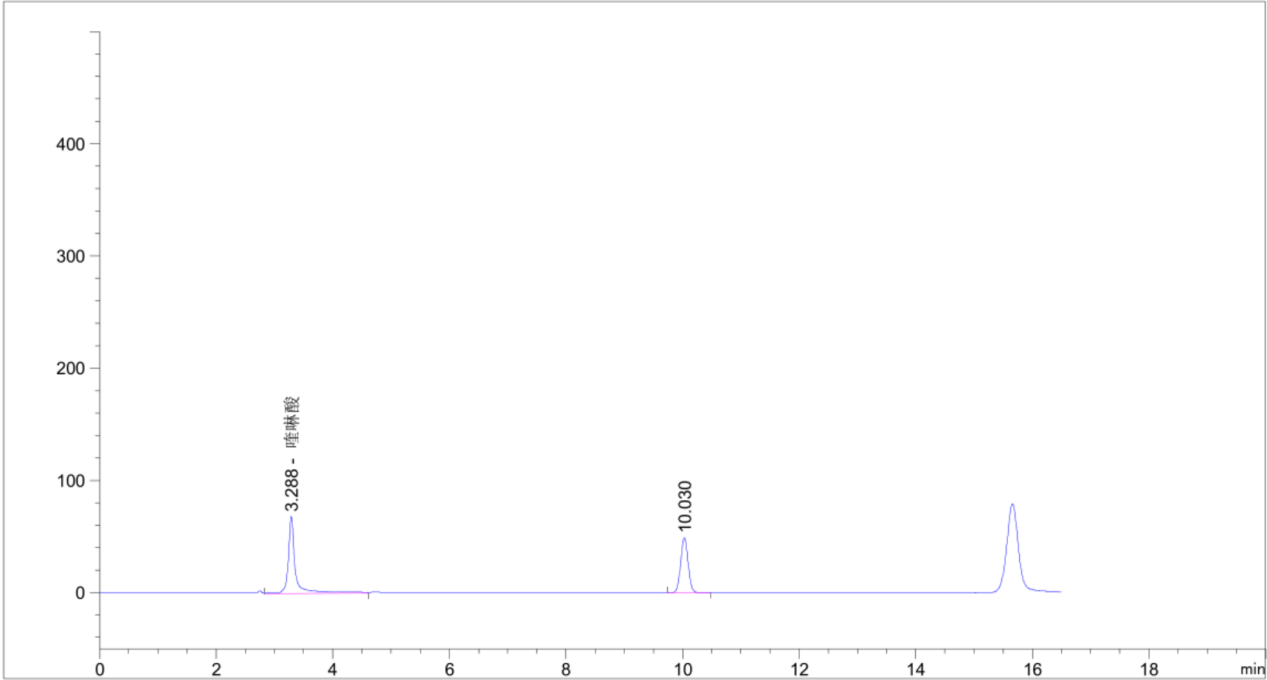


QA

QA S2


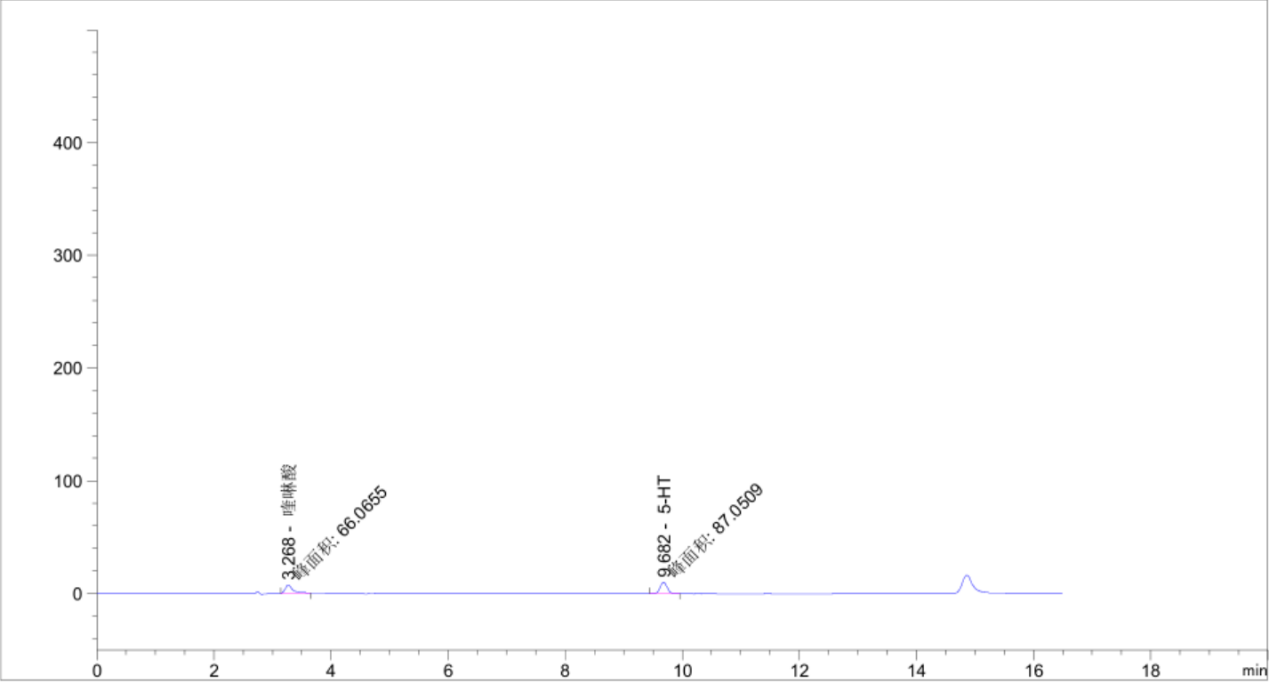


QA

QA S3


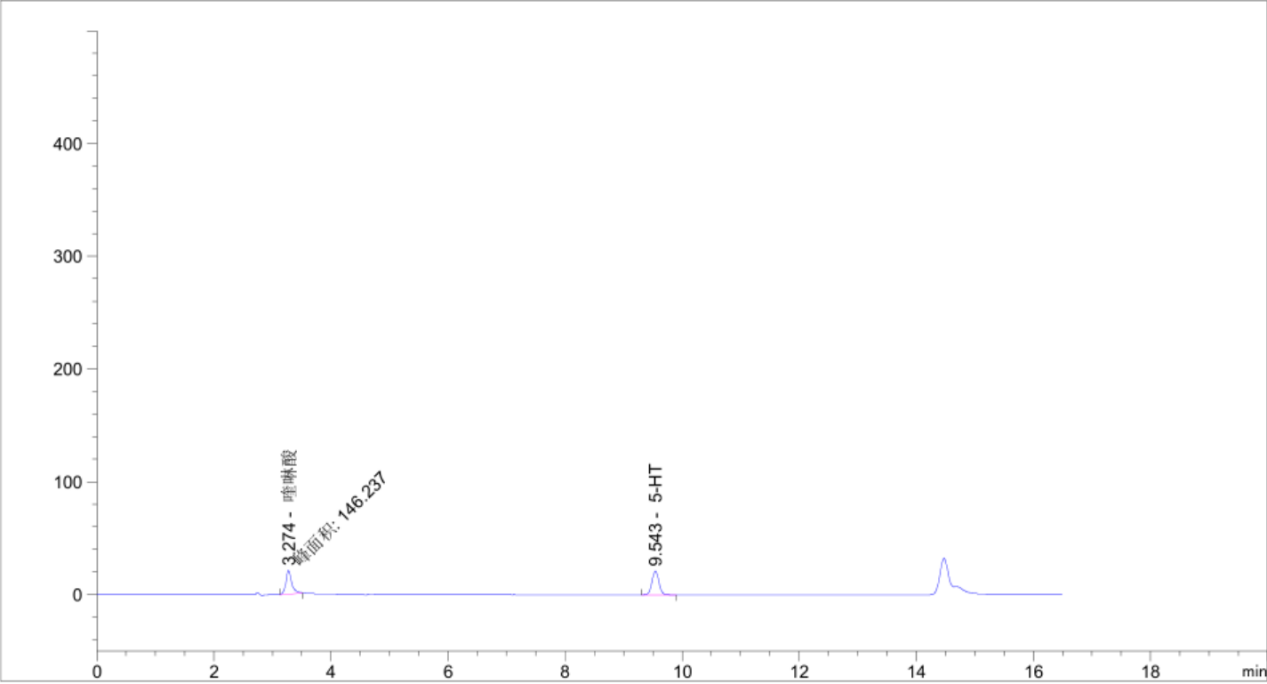


QA

QA S4


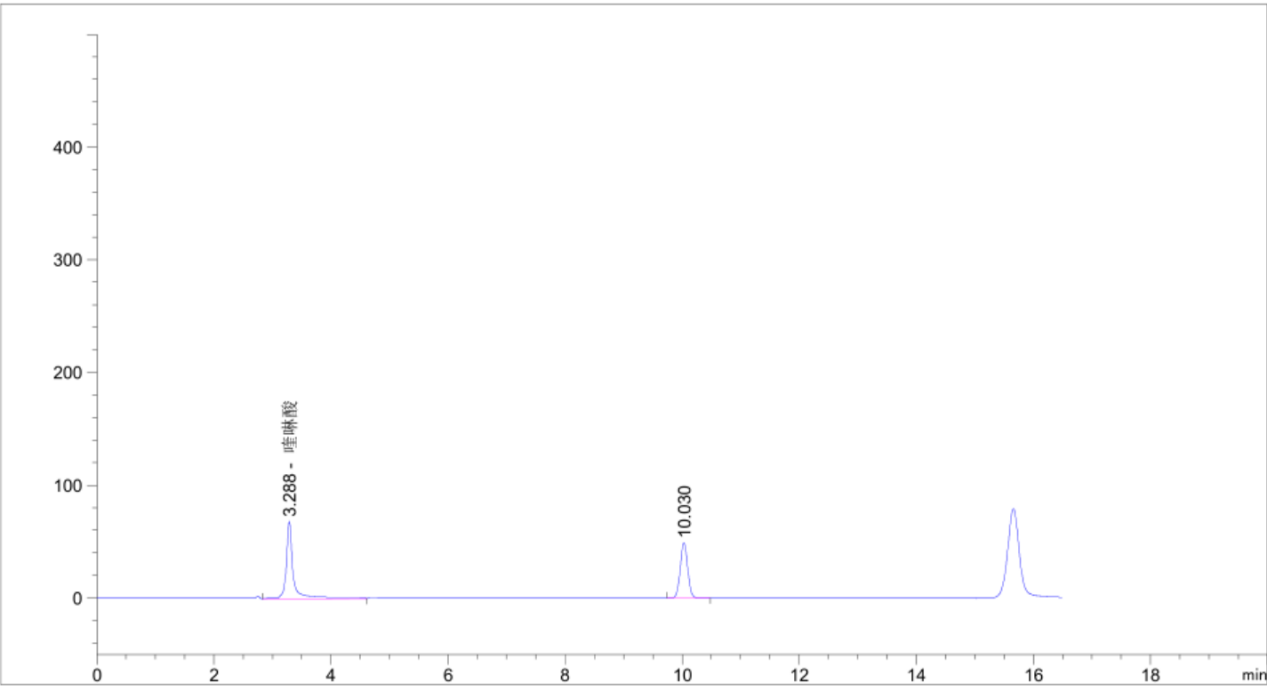


QA

QA S5


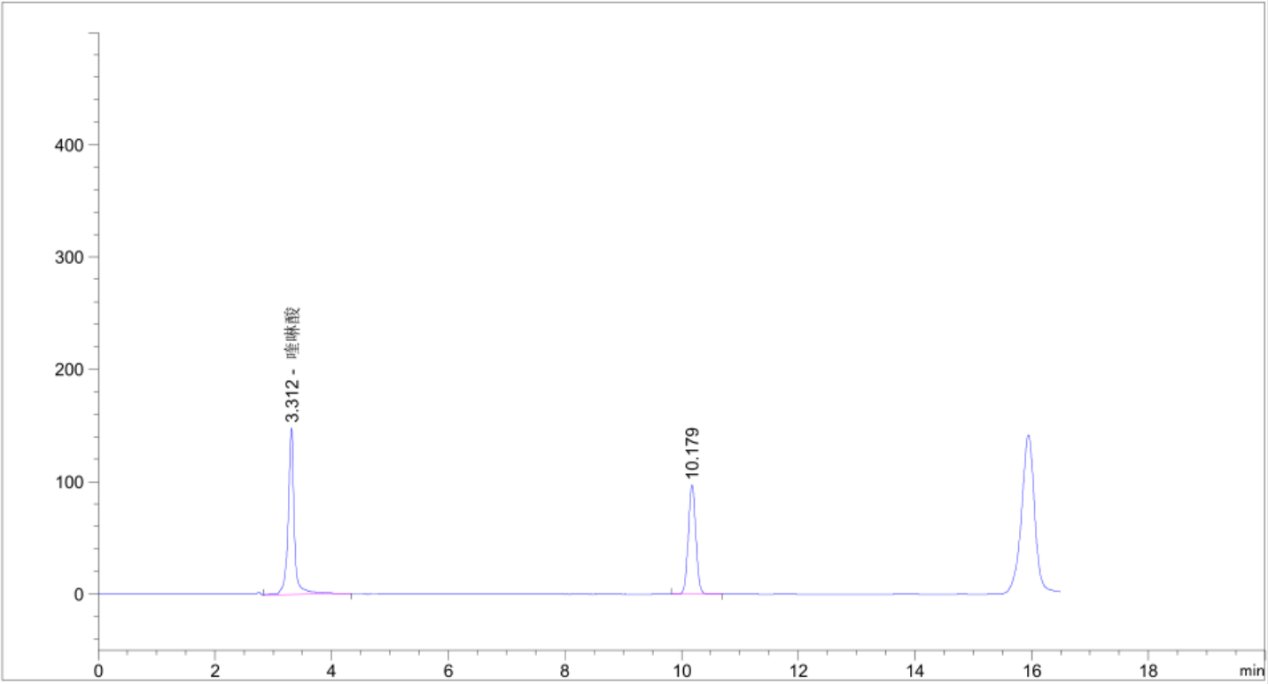


QA

QA S6


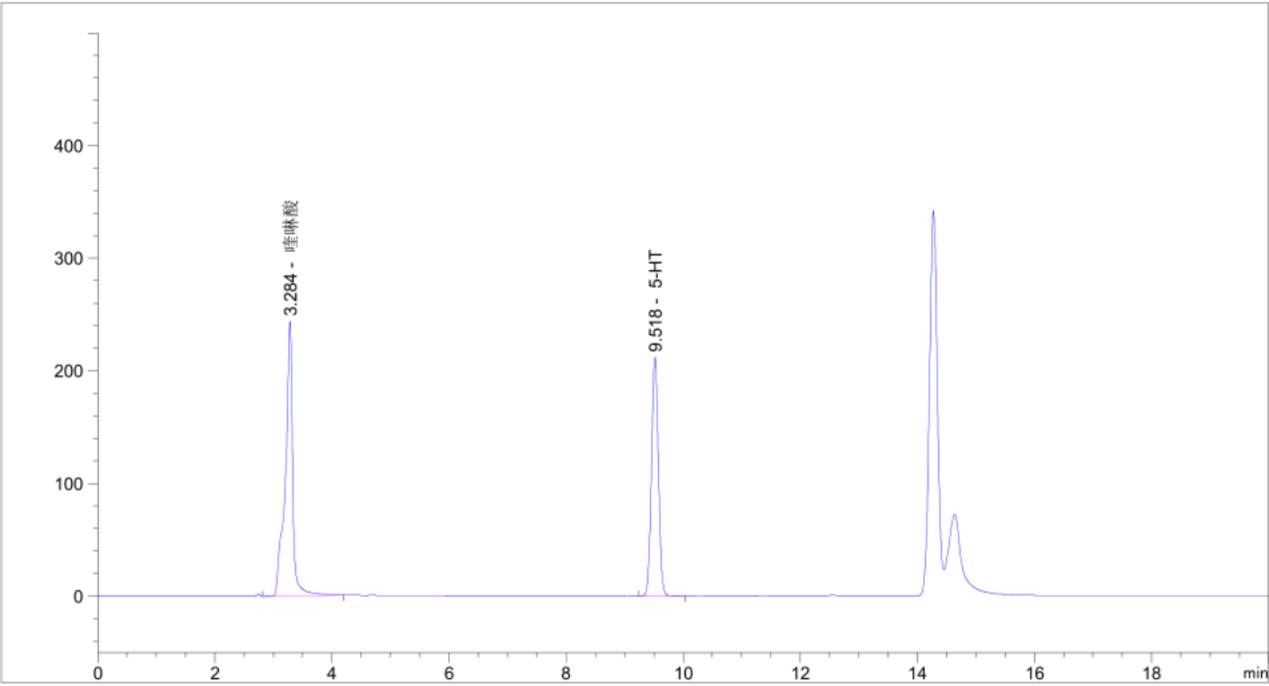


QA

KYNA S1


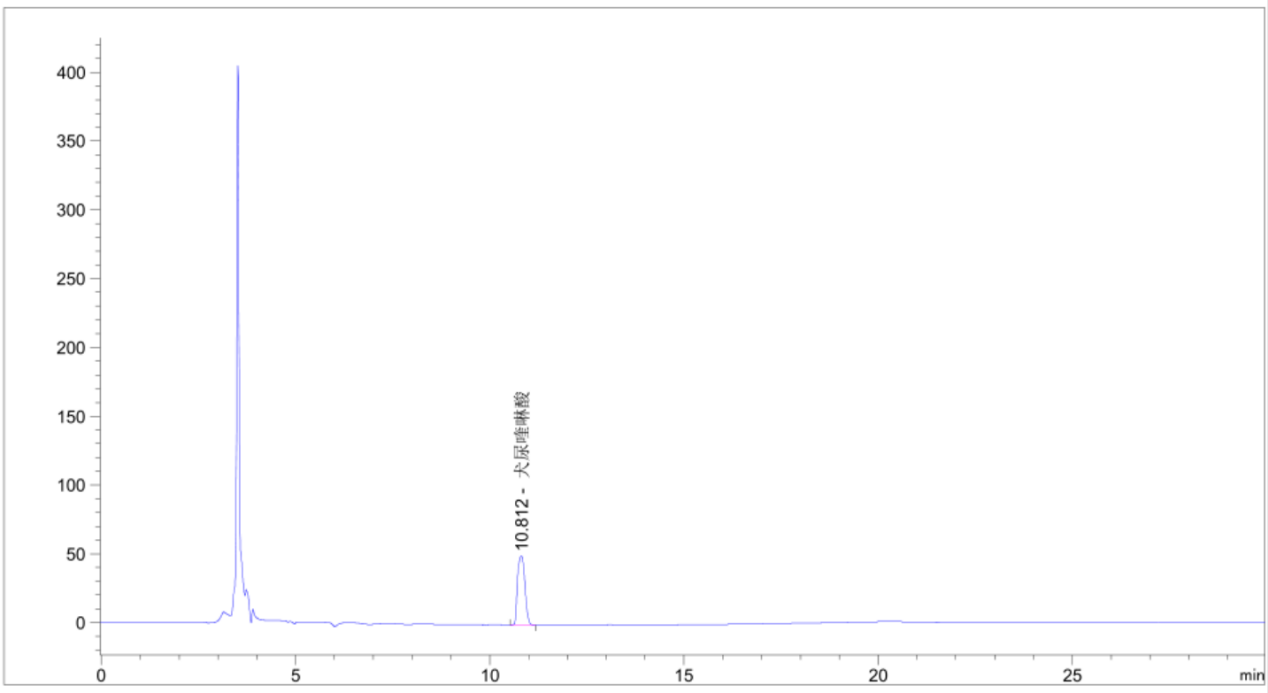


KYNA

KYNA S2


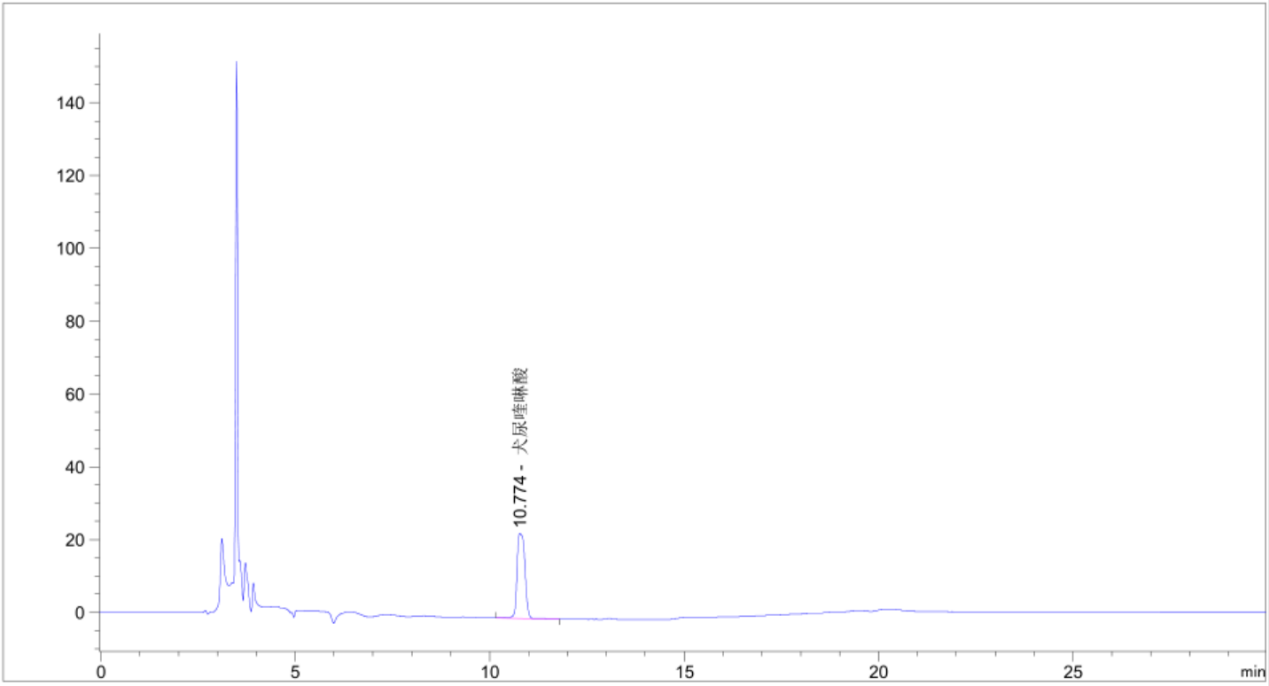


KYNA

KYNA S3


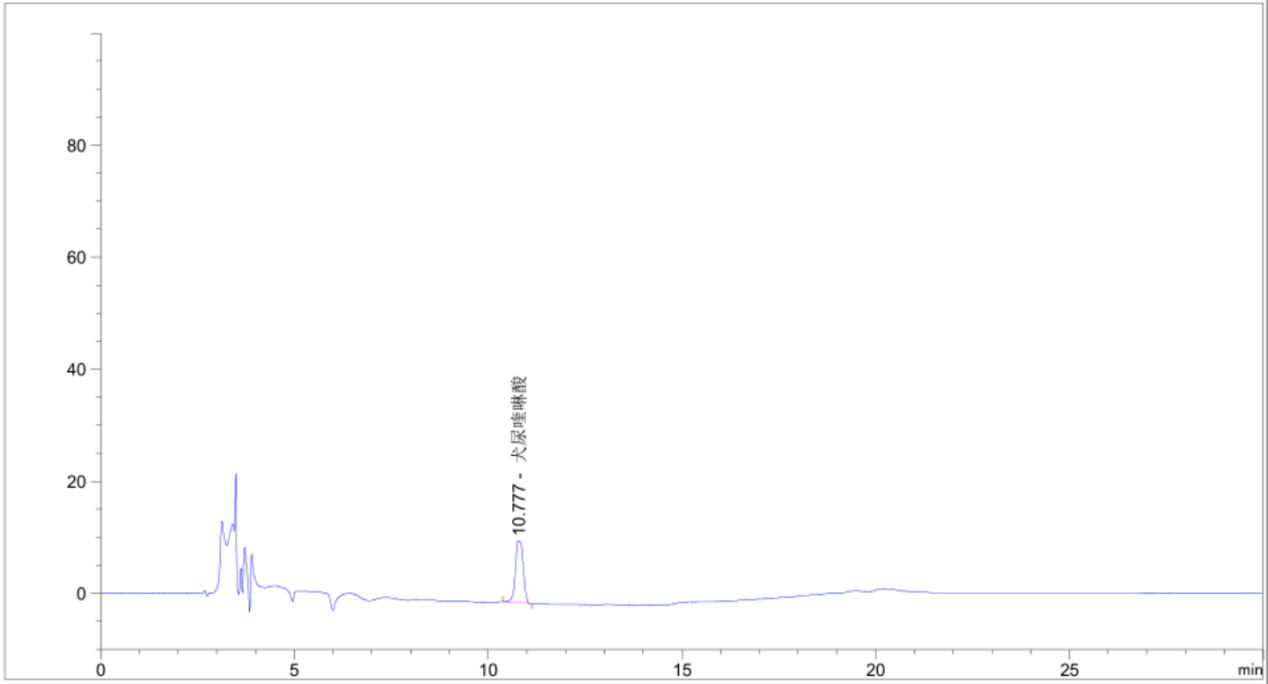


KYNA

KYNA S4


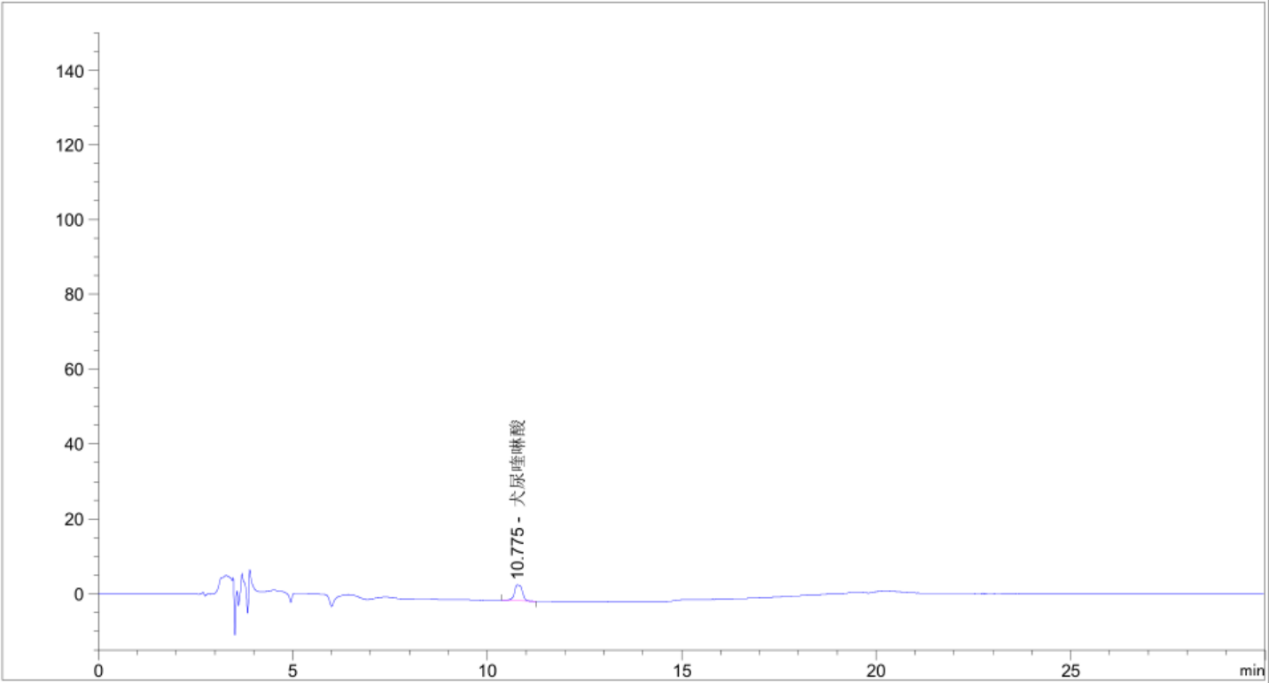


KYNA

KYNA S5


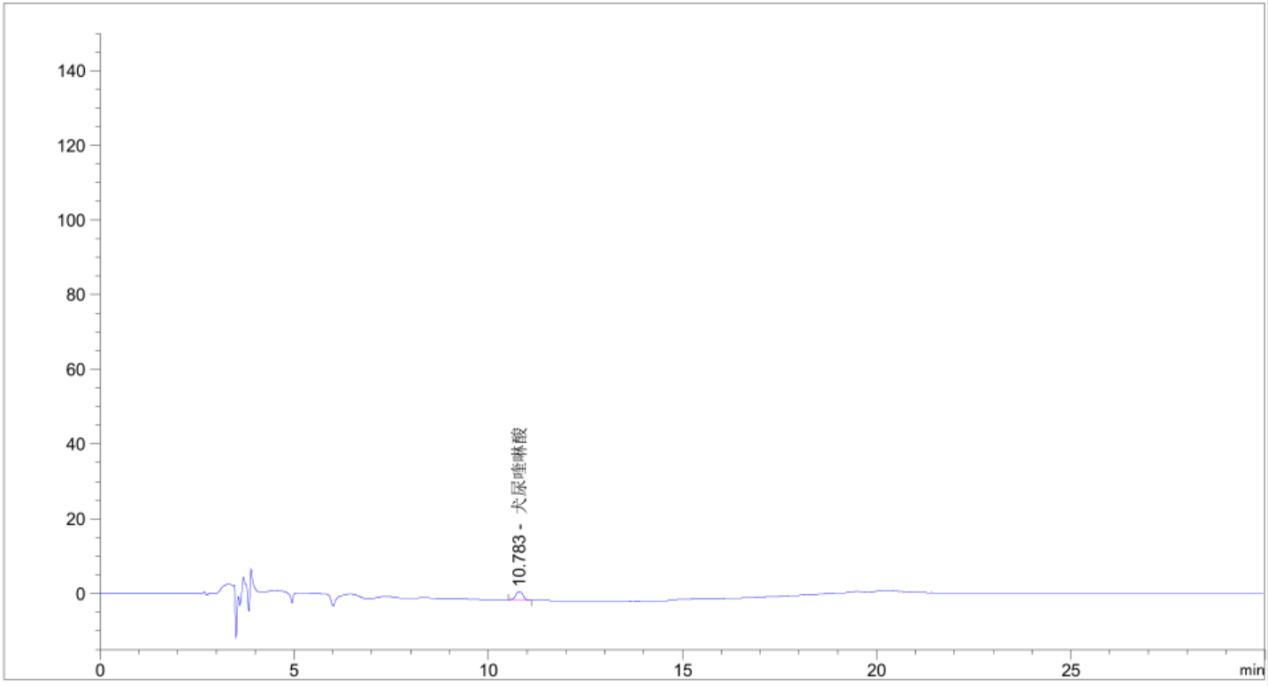


KYNA

KYNA S6


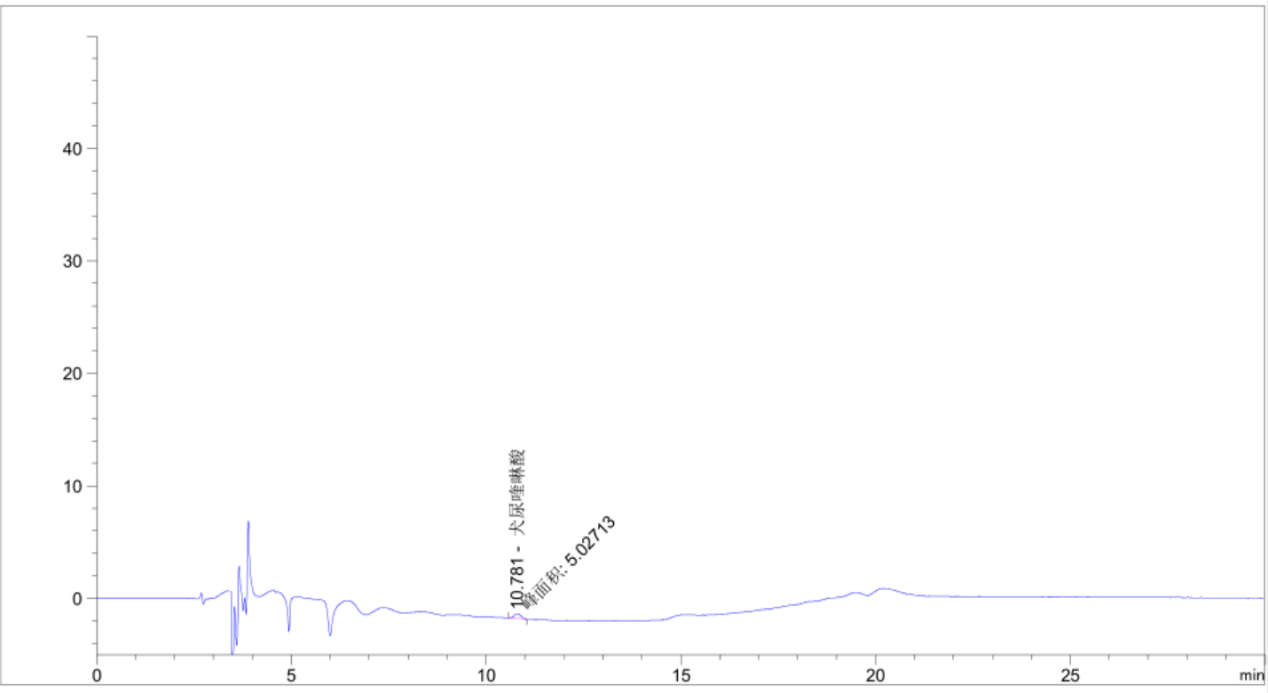


KYNA

Brain-control+PBS-1


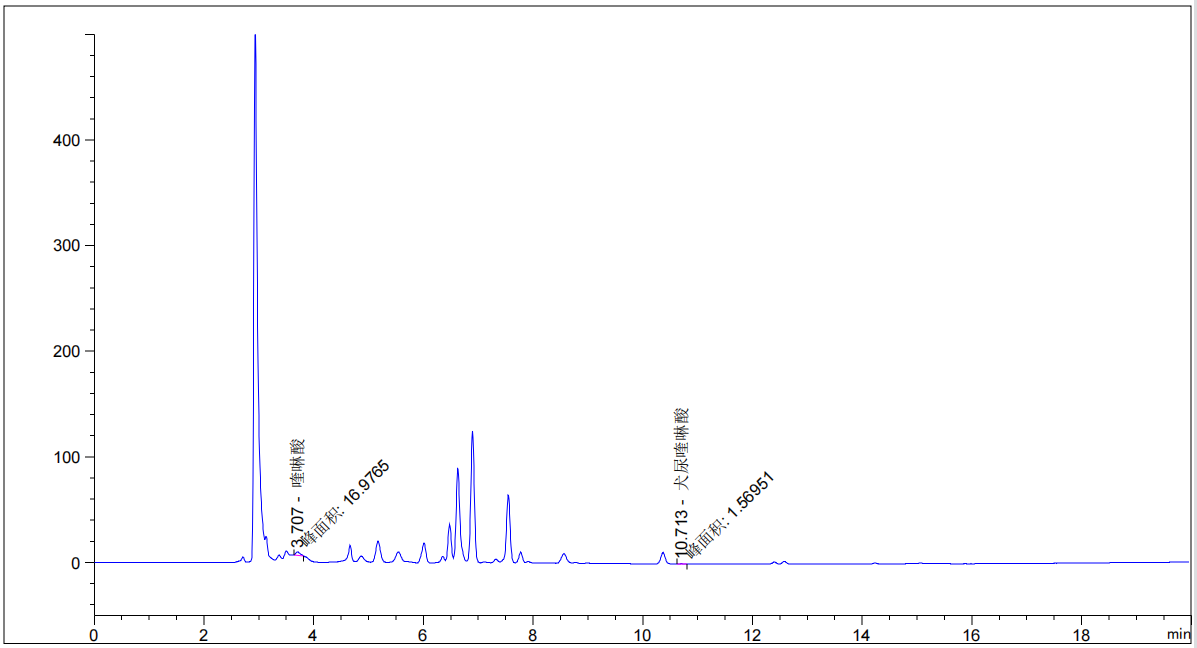


KYNA

QA

Brain-control+PBS-2


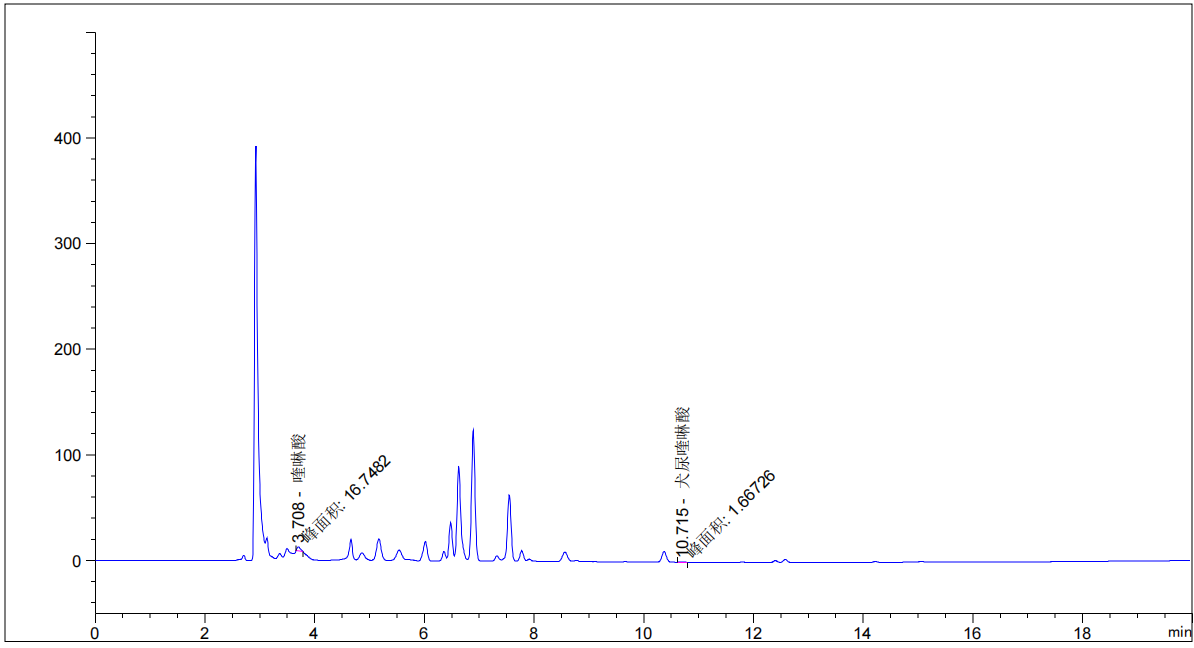


KYNA

QA

Brain-control+PBS-3


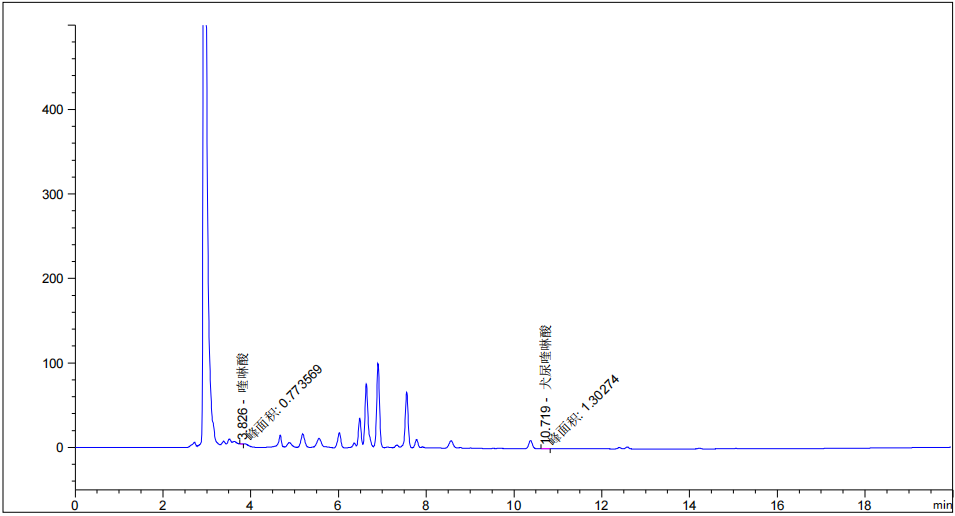


QA

KYNA

Brain-CUMS+PBS-1


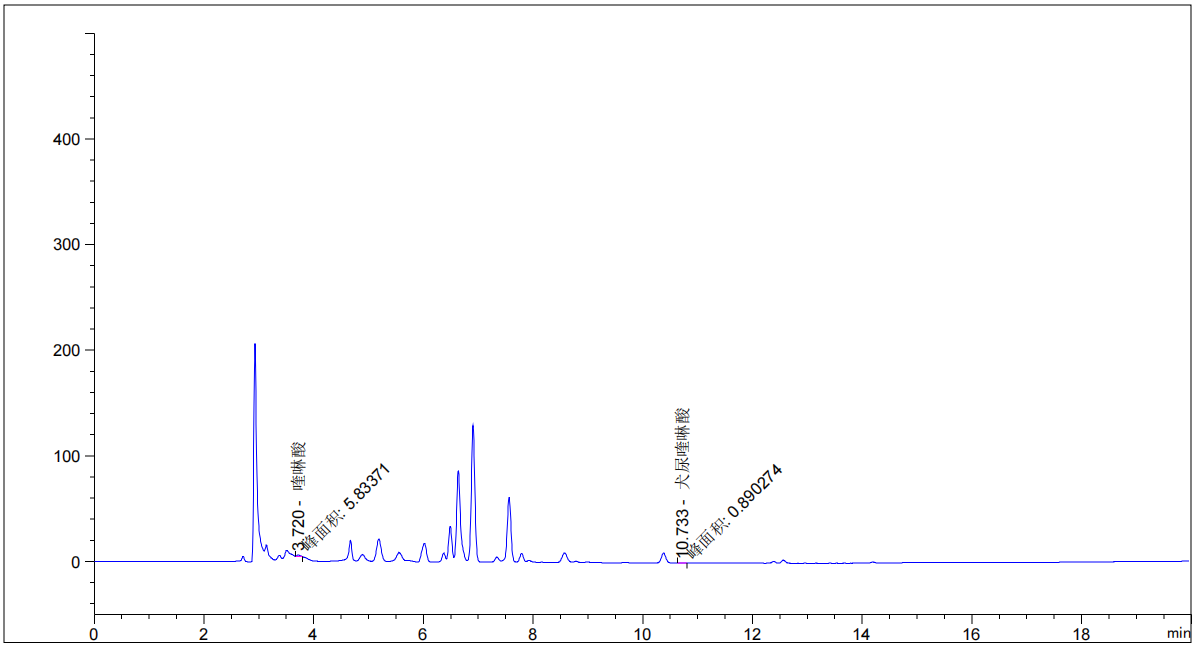


KYNA

QA

Brain-CUMS+PBS-2


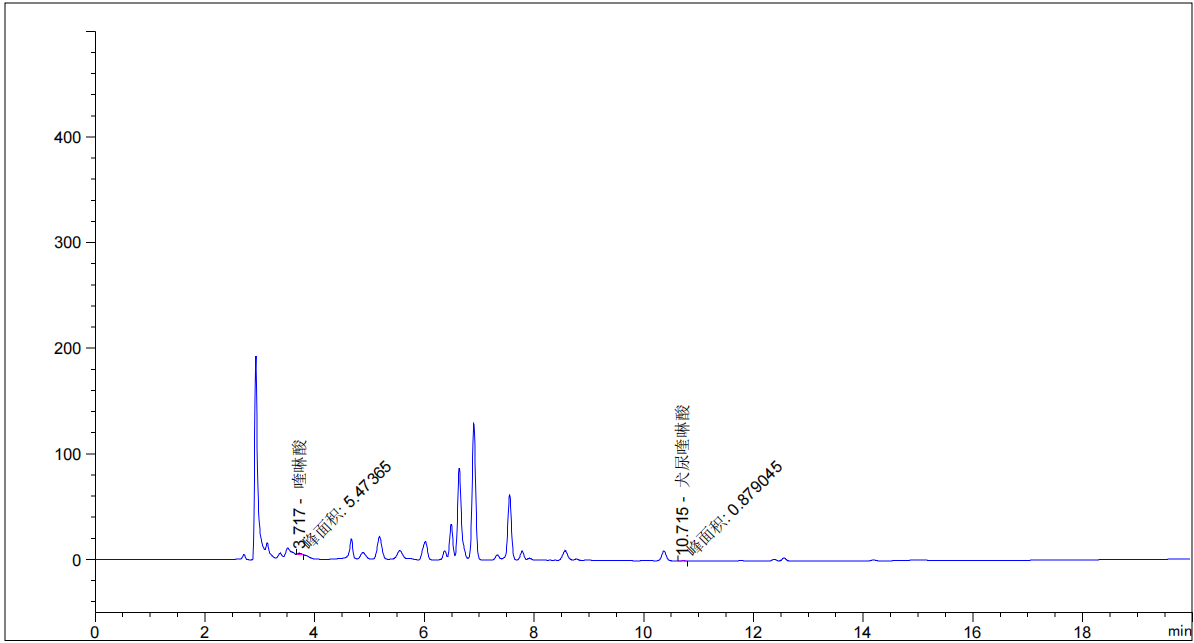


KYNA

QA

Brain-CUMS+PBS-3


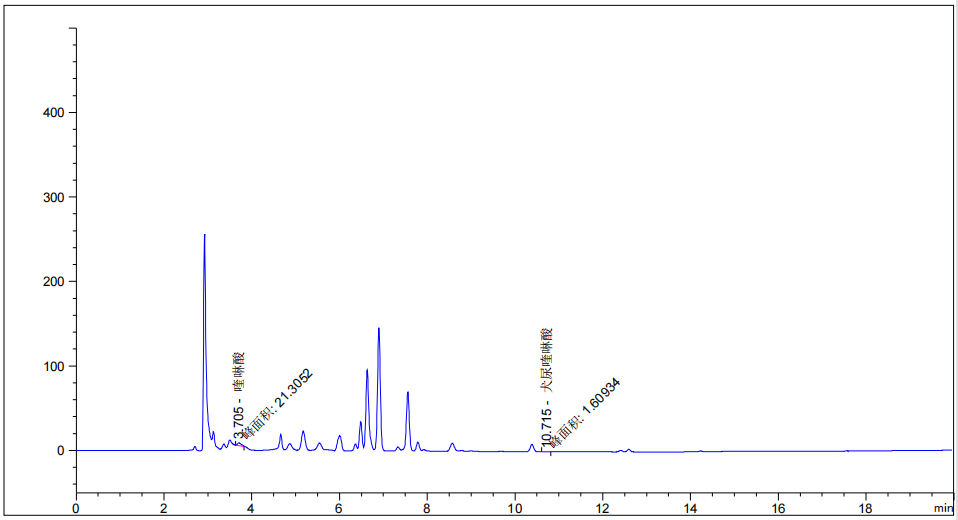


KYNA

QA

Brain-CUMS+DL-1


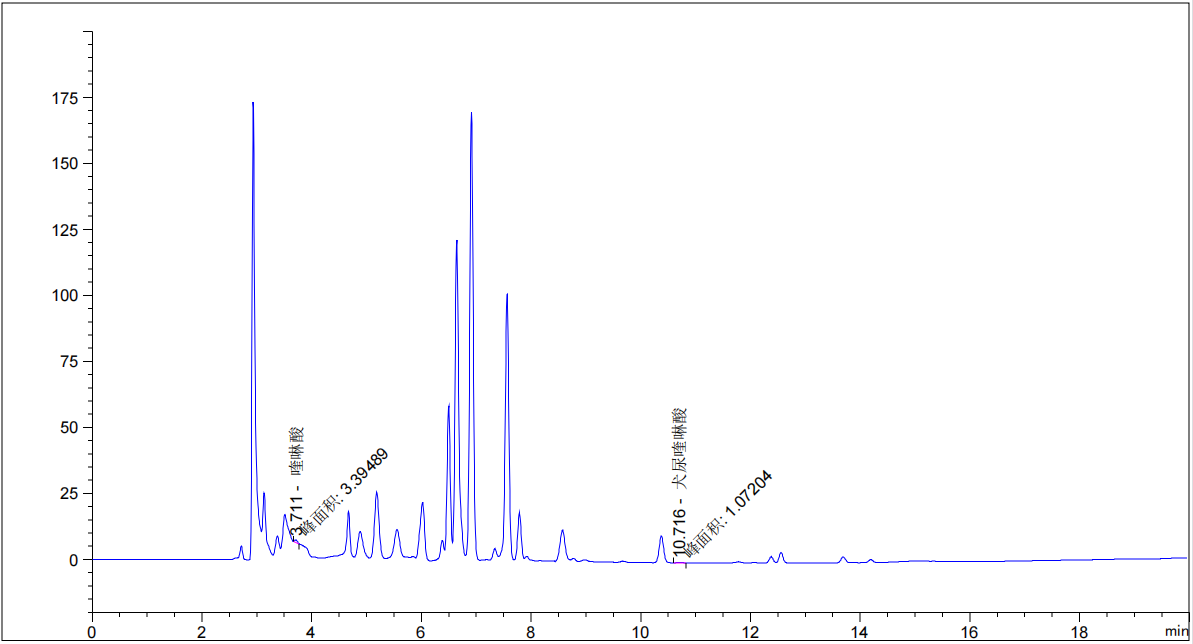


KYNA

QA

Brain-CUMS+DL-2


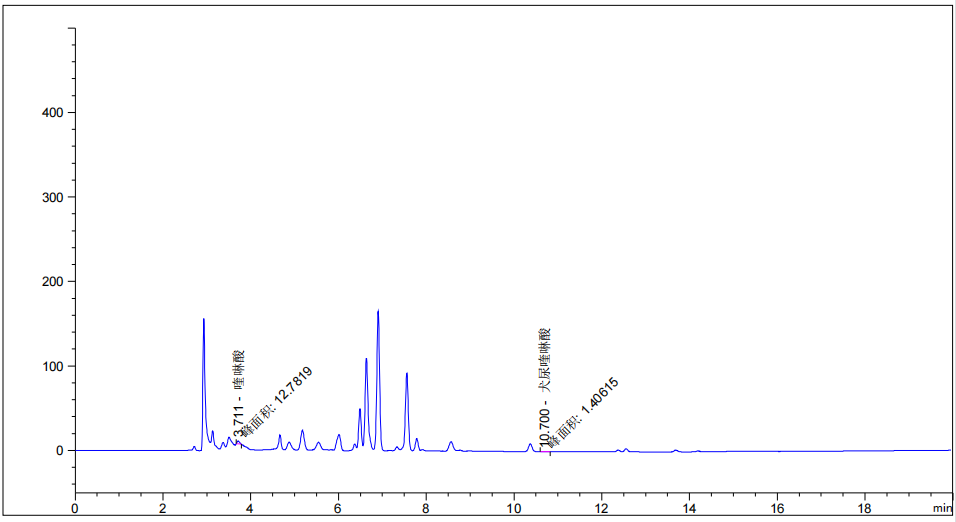


QA

KYNA

Brain-CUMS+DL-3


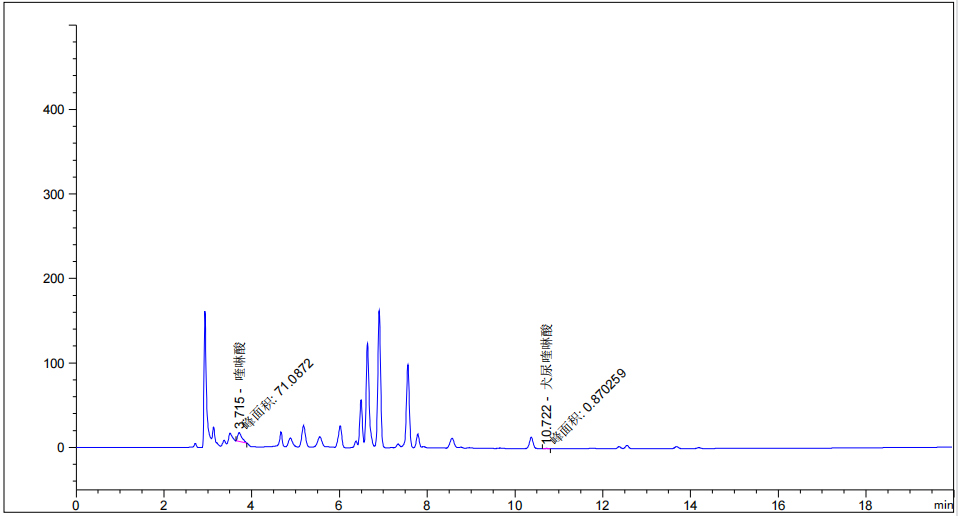


KYNA

QA

Brain-CUMS+L-1


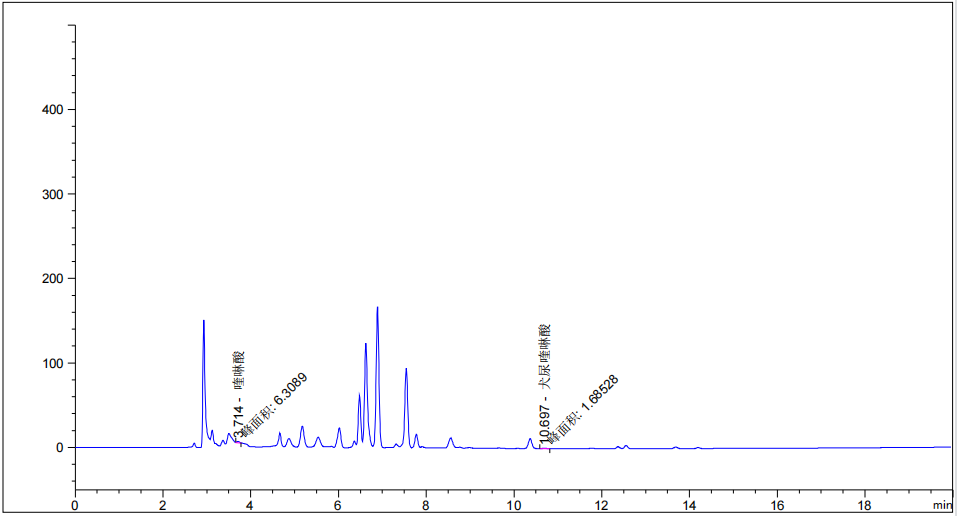


KYNA

QA

Brain-CUMS+L-2


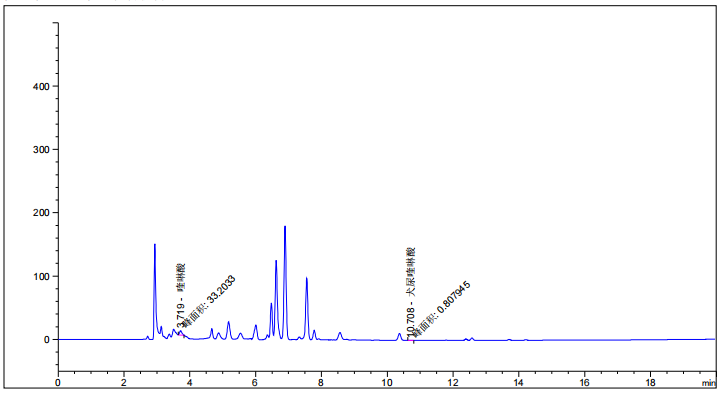


KYNA

QA

Brain-CUMS+L-3


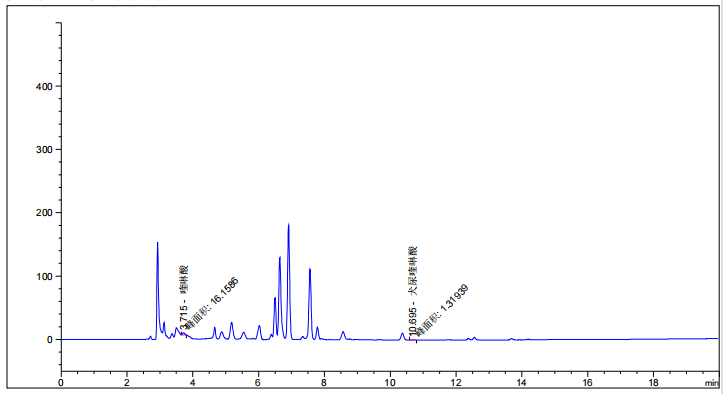


KYNA

QA

Serum-control+PBS-1


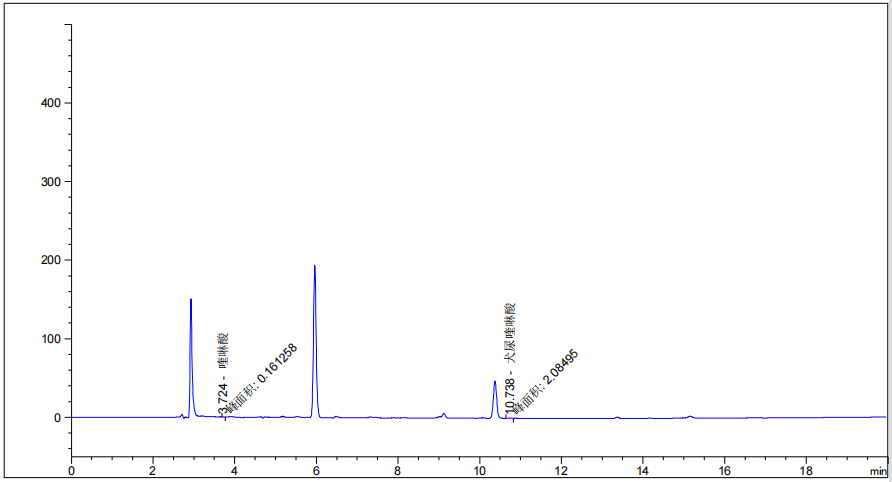


KYNA

QA

Serum-control+PBS-2


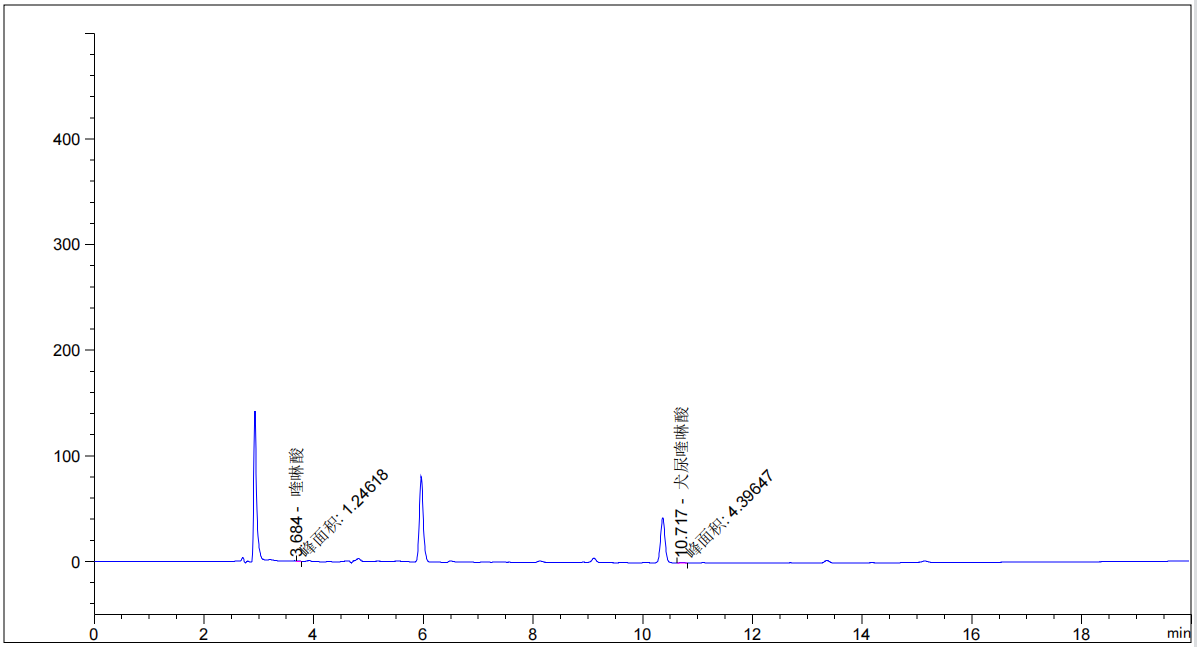


KYNA

QA

Serum-control+PBS-3


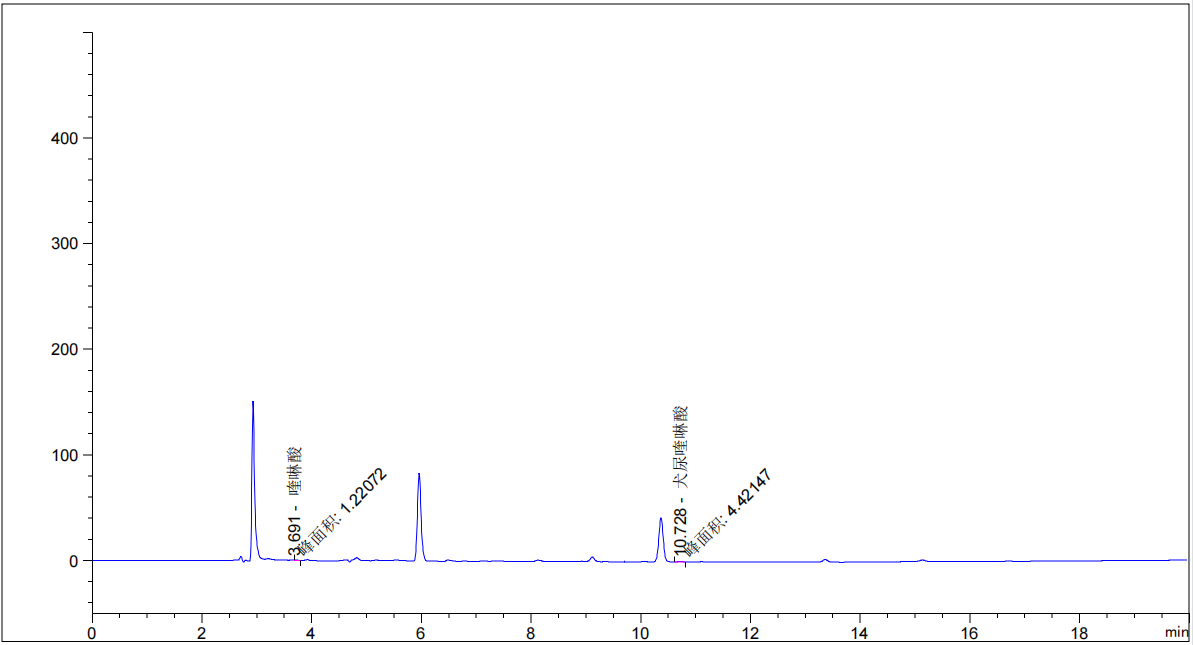


KYNA

QA

Serum-control+PBS-4


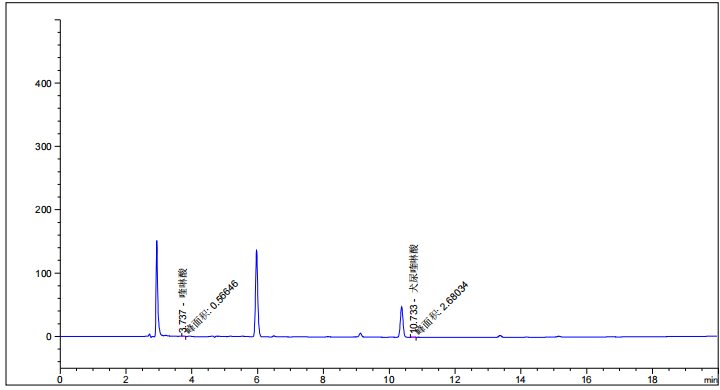


KYNA

QA

Serum-CUMS+PBS-1


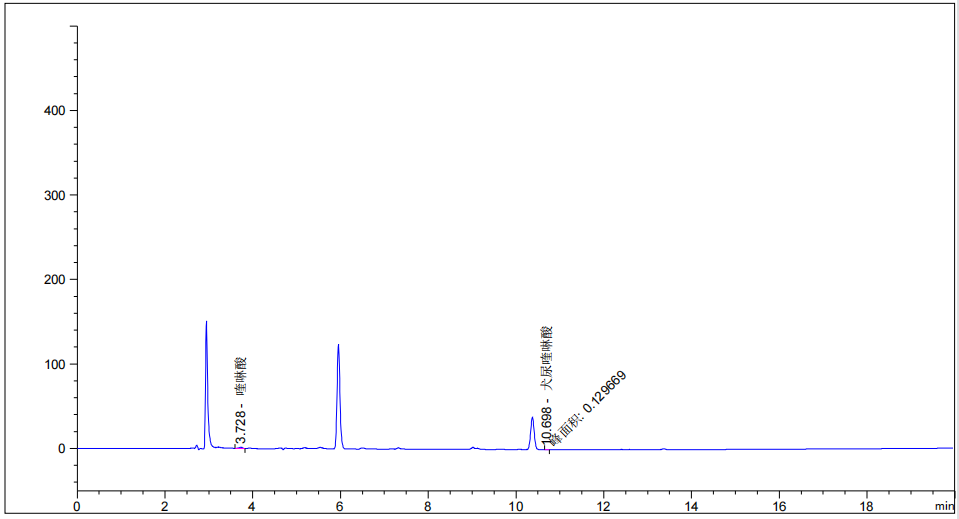


KYNA

QA

Serum-CUMS+PBS-2


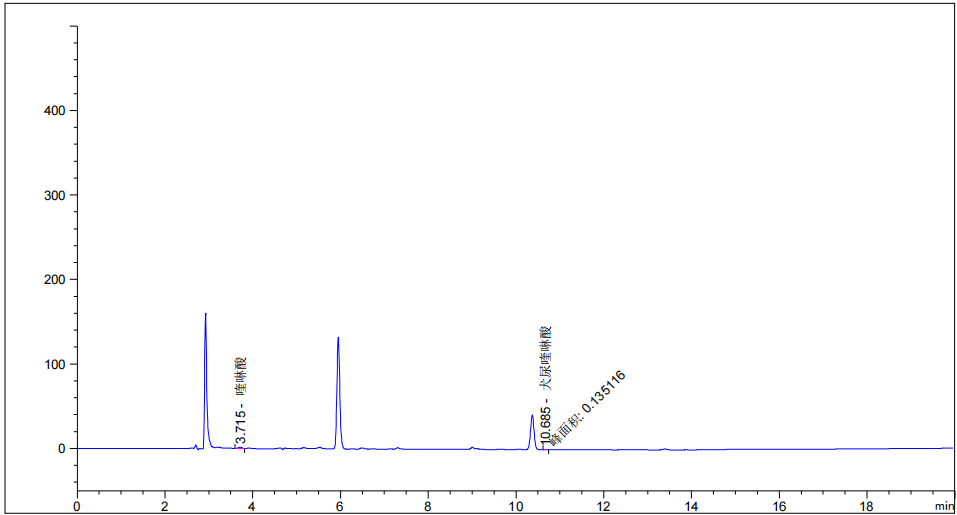


KYNA

QA

Serum-CUMS+PBS-3


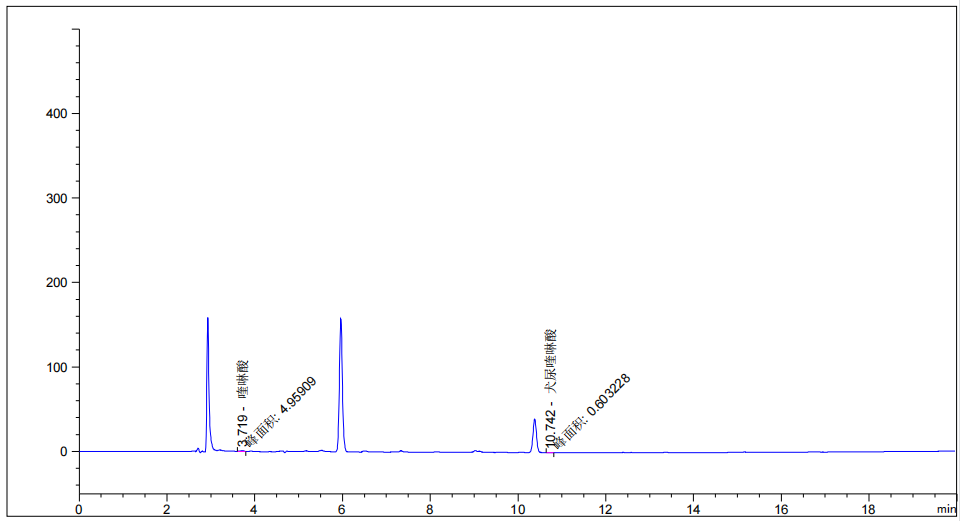


QA

KYNA

Serum-CUMS+PBS-4


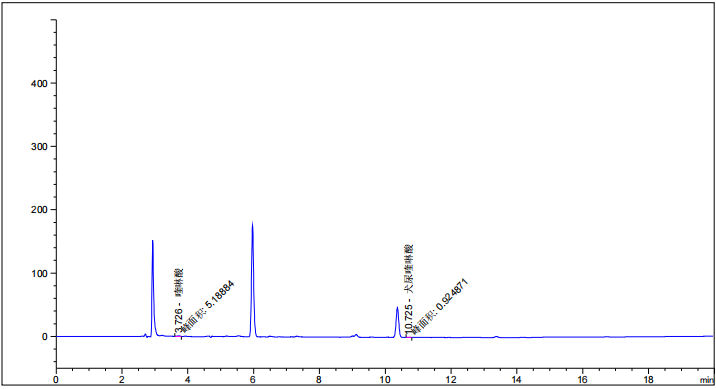


KYNA

QA

Serum-CUMS+DL-1


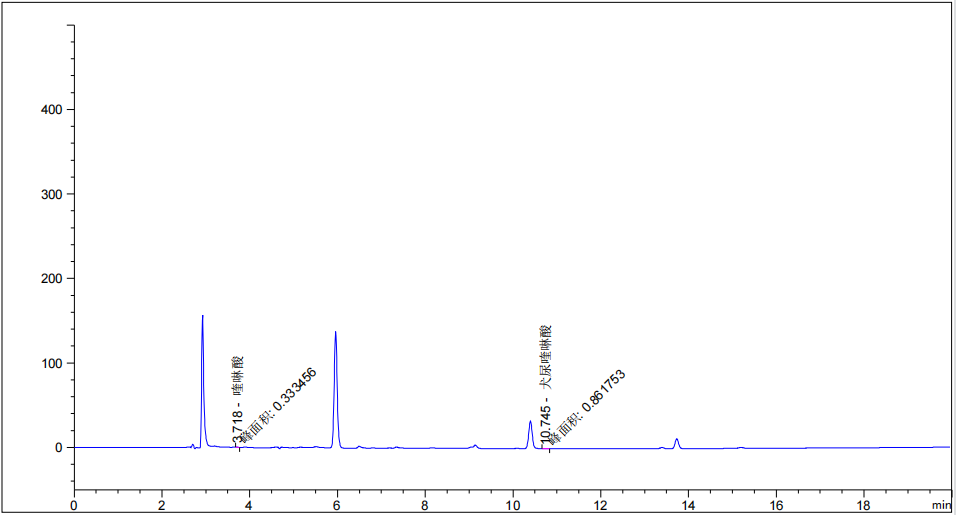


KYNA

QA

Serum-CUMS+DL-2


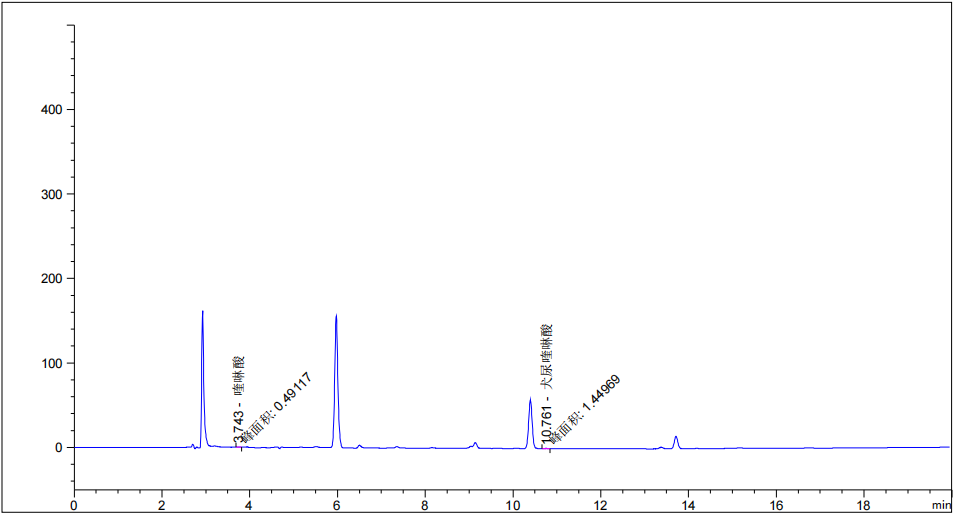


KYNA

QA

Serum-CUMS+DL-3


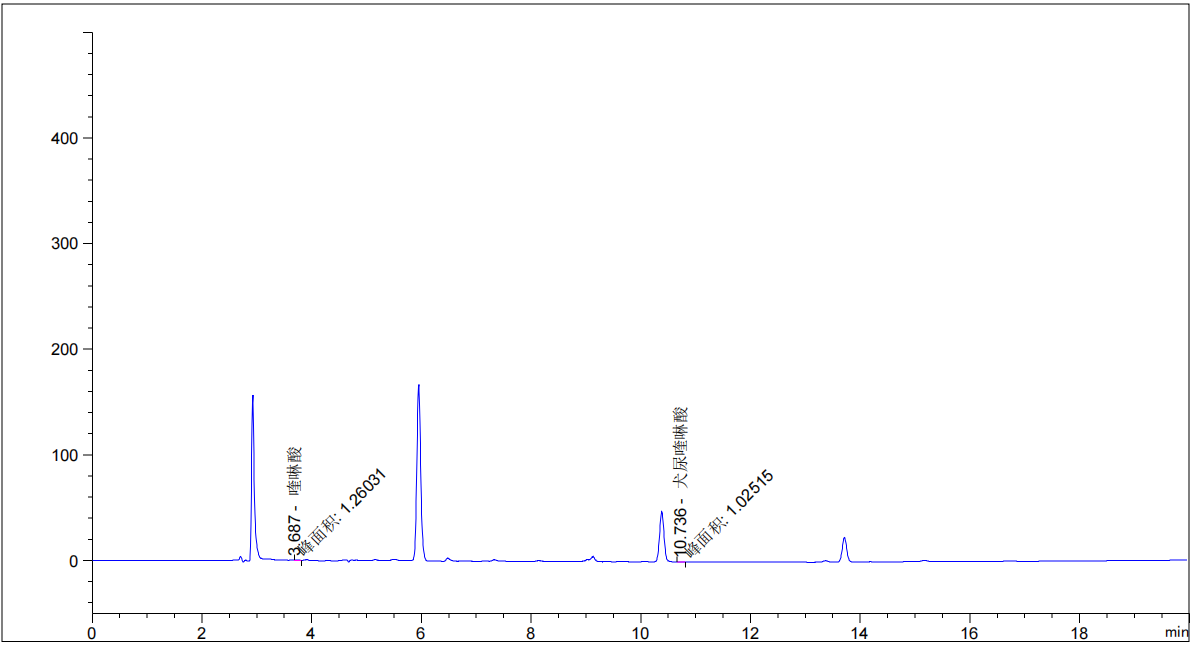


KYNA

QA

Serum-CUMS+DL-4


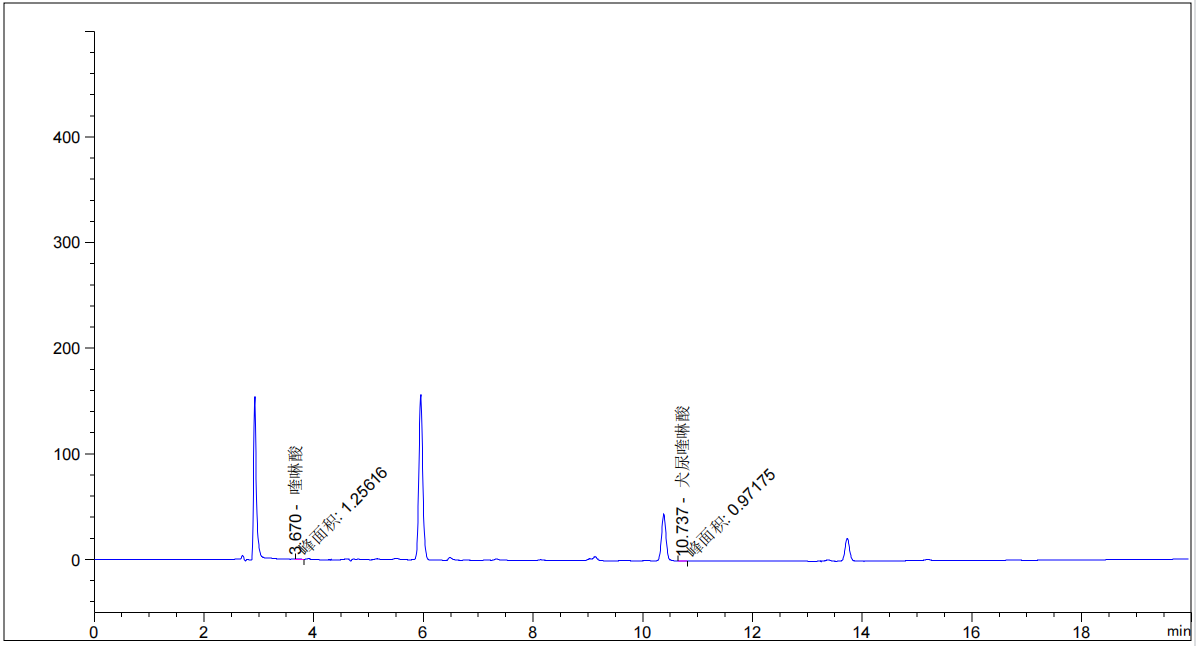


KYNA

QA

Serum-CUMS+L-1


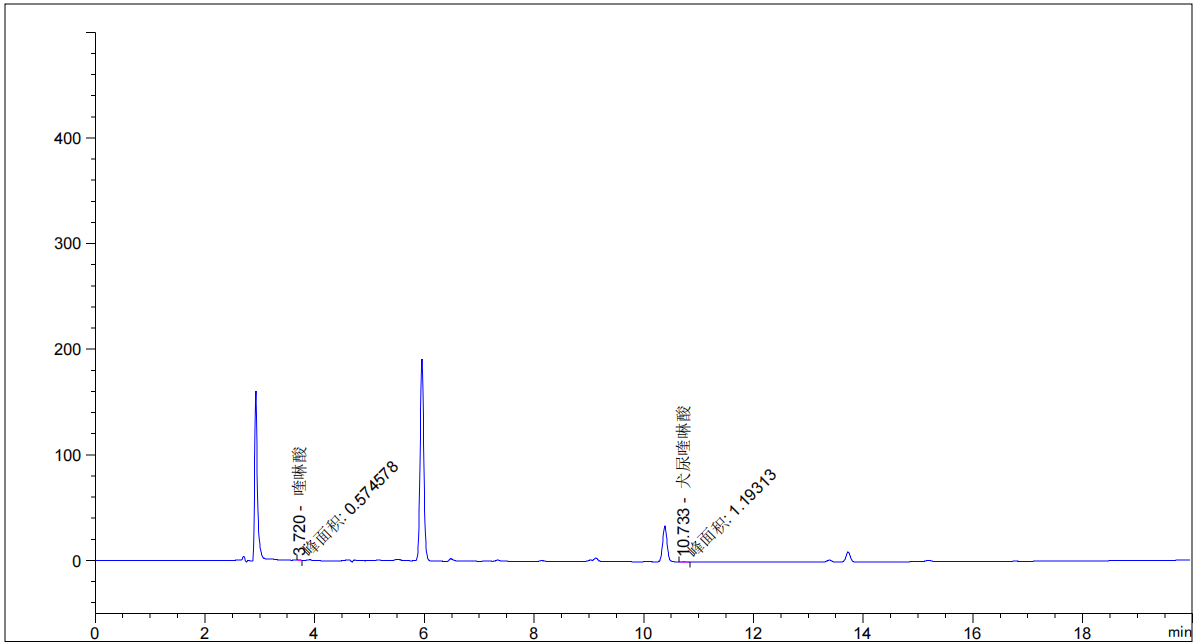


KYNA

QA

Serum-CUMS+L-2


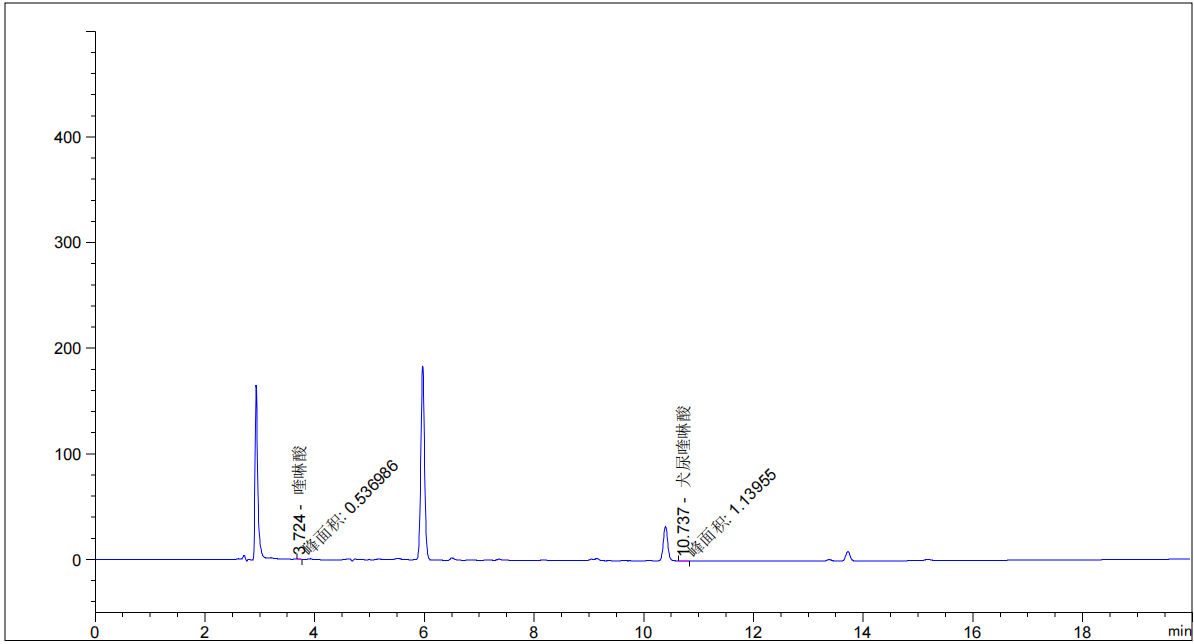


KYNA

QA

Serum-CUMS+L-3


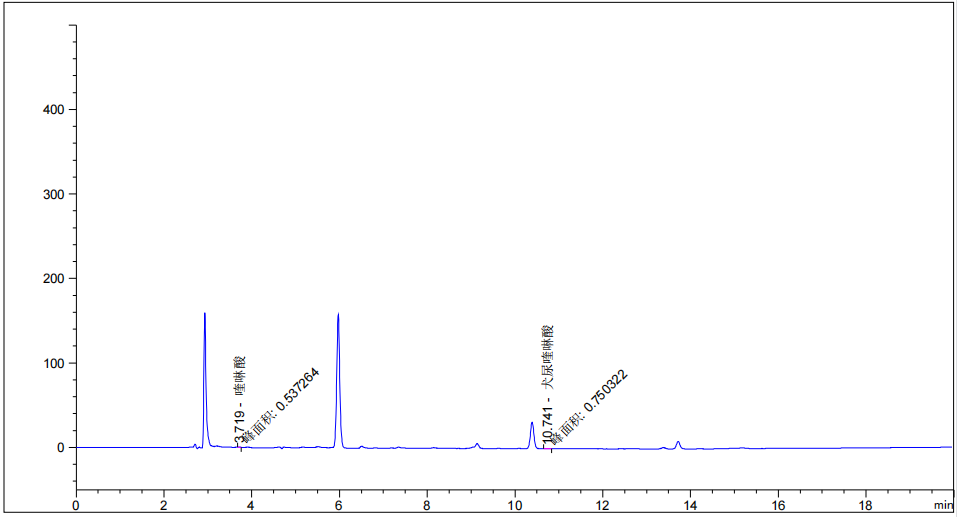


KYNA

QA

Serum-CUMS+L-4


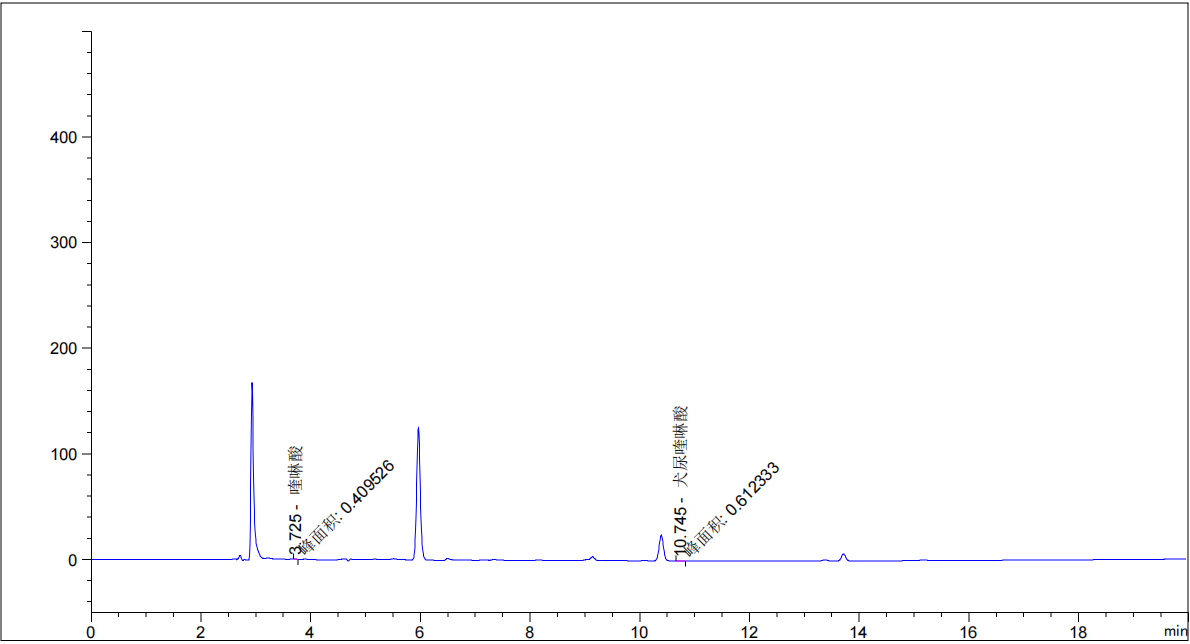


KYNA

QA
